# A Polymer‐Intercalated Modulation Assembly Strategy Towards Mesoporous Single‐Crystalline BiVO_4_ Materials for Enhanced Photocatalytic Performance

**DOI:** 10.1002/advs.202522515

**Published:** 2026-01-27

**Authors:** Wei Li, Xiaoyan Wei, Yumeng Mao, Zhengwen Tan, Wenjun Duan, Ling Zhang, Zhen‐An Qiao

**Affiliations:** ^1^ State Key Laboratory of Inorganic Synthesis and Preparative Chemistry College of Chemistry Jilin University Changchun P. R. China; ^2^ Shanxi Key Laboratory of Coal‐based Value‐Added Chemicals Green Catalysis Synthesis School of Chemistry and Chemical Engineering Shanxi University Taiyuan P. R. China; ^3^ State Key Laboratory of Supramolecular Structure and Materials College of Chemistry Jilin University Changchun P. R. China

**Keywords:** mesoporous single‐crystalline structure, photocatalytic selective oxidation, polymer‐intercalated modulation assembly, vanadium vacancy microenvironment

## Abstract

The rapid recombination of photogenerated electron–hole pairs is a bottleneck constraining the improvement of photocatalytic efficiency. The construction of porous single‐crystalline BiVO_4_ is expected to resolve this issue and provide plenty of active sites for charge carriers to promote the catalytic reaction. However, due to the fact that the synthesis process requires a delicate balance between the kinetic‐driven co‐assembly process and thermodynamic‐driven crystallization process, it faces significant challenges. Herein, a polymer‐intercalated modulation assembly strategy is proposed for synthesizing mesoporous single‐crystalline BiVO_4_ (MSC BiVO_4_) with tunable pore structure. In this case, the co‐assembly of the two metal precursors, acetate ions and polyethyleneimine (PEI), leads to the formation of an inorganic–organic composite via coordination and hydrogen bonding. Moreover, the “modulator” acetate ions obviously weaken the effect of PEI on the original crystal growth orientation of metal oligomers, thereby maintaining the single‐crystalline structure. The dendritic PEI acts as a “porogenic agent” to develop a 3D network to intercalate into metal oligomers and form the mesoporous structure. Various characterizations and theoretical calculations verified that the excellent photocatalytic performance with 99% conversion and 99% selectivity for various aromatic alcohols of the as‐prepared MSC‐BiVO_4_‐1800 is attributed to its single‐crystalline properties and well‐defined mesoporous structure with vanadium vacancy microenvironment.

## Graphical abstract

A polymer‐intercalated modulation assembly strategy was proposed to synthesize mesoporous single‐crystalline BiVO_4_ with tunable pore structure by co‐assembly of inorganic–organic composite through coordination bonds and hydrogen bonds. Thanks to single‐crystalline properties and mesoporous structure with vanadium vacancy microenvironment, the prepared BiVO_4_ photocatalyst exhibits superior selective oxidation performance for aromatic alcohols.

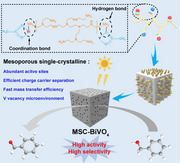

## 1 Introduction

The efficient utilization of solar energy, a clean and renewable energy source, is an important way to solve the current growing energy crisis [1, 2, 3, 4, 5]. Wherein, the design and construction of highly active semiconductor materials is vital for highly efficient solar‐driven‐energy conversion. BiVO_4_, as a class of bismuth‐based (Bi‐based) vanadate materials, has been considered as a promising candidate for semiconductor photocatalyst owing to their unique electronic structure, suitable bandgap, low cost, and availability, etc [6, 7, 8, 9]. As for BiVO_4_, the photocatalytic selective oxidation process is mainly driven by photogenerated holes (h^+^), superoxide radicals (•O_2_^−^, obtained from the reduction of molecular oxygen by photogenerated electrons), and other reactive oxygen species. Thus, achieving efficient separation and utilization of photogenerated electrons and holes is crucial to improve the photocatalytic performance for BiVO_4_, while the photogenerated electron–hole pairs generally suffer from numerous obstacles throughout the photocatalytic reaction process. Especially during the migration process, the spontaneous recombination of photogenerated electron–hole pairs leads to unsatisfying catalytic efficiency [10, 11, 12, 13].

As a conventional strategy, imparting single‐crystalline properties to Bi‐based salts can eliminate grain boundaries that act as recombination centers of photogenerated carriers during the migration process. But low specific surface area and fewer active sites of common bulk single‐crystalline Bi‐based salt materials result in the fact that the photogenerated electron–hole pairs have insufficient access to the active sites to participate in the photocatalytic reaction. In order to further resolve the issue, the fabrication of porous single‐crystalline Bi‐based salts can provide more adsorption and photocatalytic active sites with both efficient separation and fast transport of photogenerated carriers [14]. Moreover, the introduction of a porous structure can also facilitate the exposure of more surface defects and enhance the diffusion and transport of reactants and products, which is of great importance for optimizing photocatalytic activity [15, 16, 17]. However, the rational synthesis of porous single‐crystalline Bi‐based salt materials faces great challenges. This is mainly attributed to the fact that the kinetic‐driven co‐assembly process between metal precursors and pore templates is difficult to be well compatible with the thermodynamic‐driven crystallization process [14, 18]. Specifically, the fast and inconsistent hydrolysis and condensation kinetics of Bi^3+^ and V^5+^ precursors are difficult to control well, as well as the kinetic‐driven interaction and co‐assembly process between inorganic precursors and pore template, leading to the formation of dense non‐porous materials. In addition, the crystal growth along the specific orientation during high‐temperature crystallization is susceptible to destroying the porous structure.

Herein, a polymer‐intercalated modulation assembly strategy was proposed to construct the mesoporous single‐crystalline BiVO_4_ (MSC BiVO_4_) materials. Plenty of acetate ions can coordinate with the two metal precursors to form complexes and modify the hydrolysis and condensation kinetics of Bi^3+^ and V^5+^ precursors. Besides, the aforementioned complexes interact with polyethyleneimine (PEI) through coordination bonds and hydrogen bonds, respectively, thereby forming an inorganic–organic composite. During this process, the acetate ions act as a “modulator”, not only modulating and matching the interaction between PEI and the two metal precursors but also weakening the influence of PEI on the original single‐crystalline growth orientation of metal oligomers. The dendritic PEI, as a “porogenic agent”, can form a 3D spatial network during the synthesis process, which are able to be well intercalated into the metal oligomers, and endows a suitable space for crystallization and growth of Bi^3+^ and V^5+^ metal oligomers, thereby promoting the formation of a mesoporous structure. Moreover, by adjusting the molecular weight of PEI (M_w_ 300–70000), the intercalation effect on metal oligomers during the growth and crystallization process was regulated, and a series of 3D MSC BiVO_4_ materials were synthesized with tunable pore wall thickness and pore structure. The prepared MSC BiVO_4_, synthesized by using the PEI molecular weight of 1800 (MSC‐BiVO_4_‐1800) as a photocatalyst, exhibits superior selective oxidation performance (99% conversion and 99% selectivity) for various aromatic alcohols. Combining the theoretical density functional theory (DFT) calculations and various characterizations, the great photocatalytic activity can be ascribed to the single‐crystalline properties without a grain boundary that inhibits the recombination of photogenerated electron–hole pairs and accelerates carrier transport efficiency. Furthermore, the well‐defined mesoporous structure not only provides plenty of active sites and boosts mass transfer efficiency, but also facilitates the construction of a vanadium vacancy microenvironment, thereby promoting the broadening of the visible light adsorption range, the activation of benzyl alcohol (BA), and the separation of charge carriers, which is conducive to the photocatalytic selective oxidation of BA.

## 2 Results and Discussion

**FIGURE 1:**
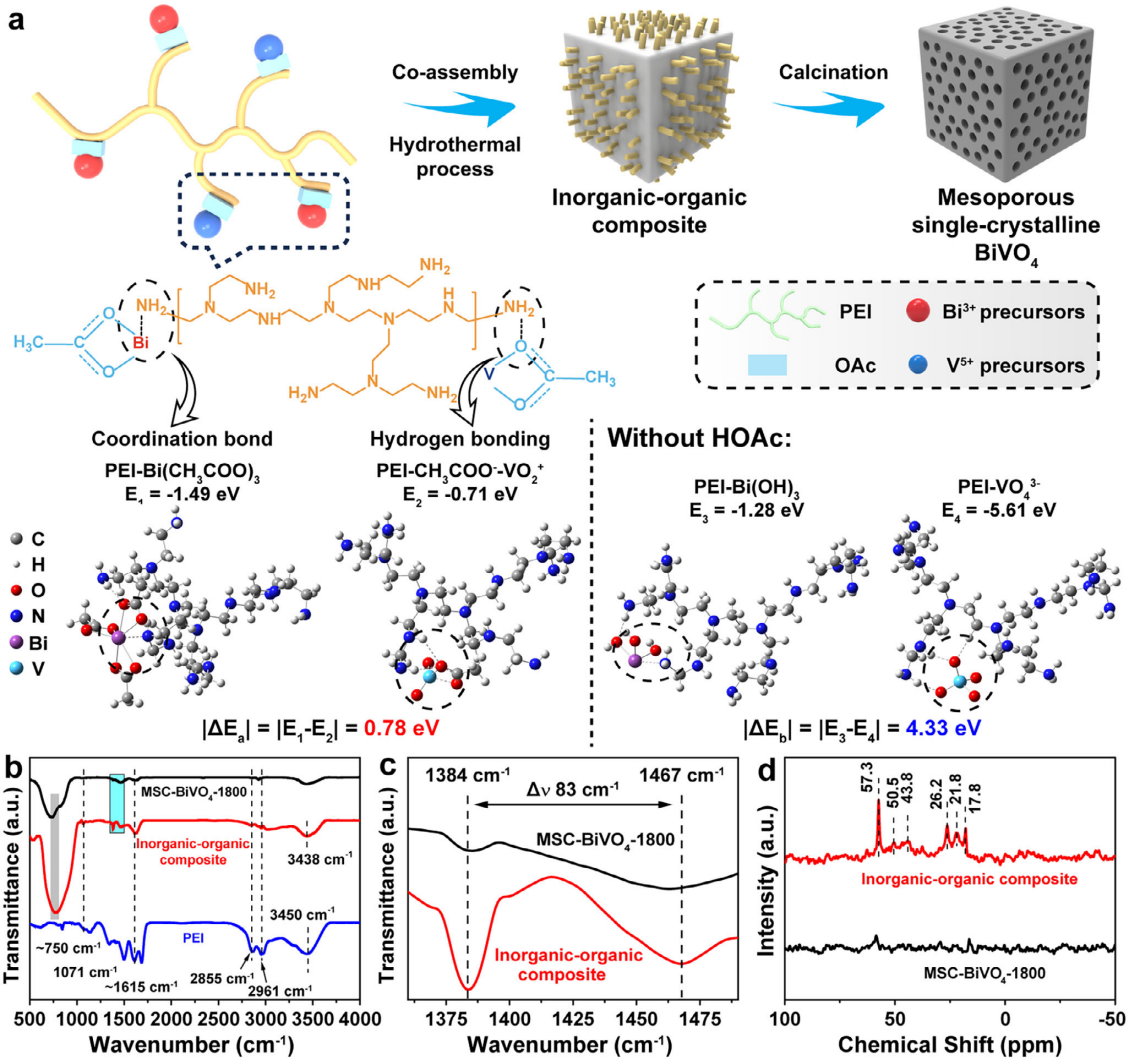
(a) Schematic illustration of the formation process for MSC BiVO_4_ materials by polymer‐intercalated modulation assembly strategy and DFT calculations of the binding energy between PEI and metal precursors without and with HOAc addition; (b) FT‐IR spectra, (c) Local region enlarged FT‐IR spectra and (d) NMR spectra of MSC‐BiVO_4_‐1800 and PEI/HOAc/Bi^3+^/V^5+^ inorganic–organic composite.

The schematic illustration of the synthesis for MSC BiVO_4_ by polymer‐intercalated modulation assembly strategy is shown in Figure 1a. In this strategy, PEI, HOAc, Bi^3+^, and V^5+^ metal salts are dissolved in aqueous solution in turn at the early stage. The amino groups in PEI greatly promote the ionization of HOAc, and the resulting large amount of acetate ions coordinate with metal ions through the bidentate mode, which is verified by the Fourier transform‐infrared (FT‐IR) characteristic peaks of asymmetrical υ^as^(COO^−^) and symmetrical υ^s^(COO^−^) stretching vibrations at 1467 and 1384 cm^−1^, respectively, for the PEI/HOAc/Bi^3+^/V^5+^ inorganic–organic composite with the frequency difference of 83 cm^−1^ (Figure 1b,c; Figures S1 and S2) [19, 20]. Besides, such coordination interactions can also effectively alleviate the hydrolysis and condensation rates of the metal precursors, facilitating the subsequent co‐assembly process. Furthermore, the FT‐IR peak attributed to the ─NH group shows an obvious red shift for the inorganic–organic composite (3438 cm^−1^) compared to PEI (3450 cm^−1^), manifesting the formation of hydrogen bonds between PEI and metal oligomers. The FT‐IR peaks located at 1071, 1615, 2855, and 2961 cm^−1^ are indexed to the stretching vibrations of C─N bonds, NH groups, and methylene groups of PEI, respectively [21, 22, 23]. In addition, the peaks at 43.8, 50.5, and 57.3 ppm in the ^13^C nuclear magnetic resonance (NMR) spectra (Figure 1d) are also assigned to the primary, secondary, and tertiary amine groups of PEI, respectively [24, 25]. The characteristic peaks belonging to PEI in the FT‐IR and NMR spectra disappeared after calcination at 350°C, verifying the presence of PEI in the inorganic–organic composite. Thus, the above spectral characterizations confirm the formation of an inorganic–organic composite and the co‐assembly of metal precursors, acetate ions, and PEI by coordination bonds and hydrogen bonds.

For exploring the vital role of acetate ions as a “modulator” in the inorganic–organic composite (Figures S3 and S4 and Table S1), the DFT theoretical calculations (the lower part of Figure 1a) on the binding energy between PEI, acetate ions, and metal precursors with and without the introduction of HOAc during the synthesis process were conducted. With the introduction of HOAc, the acetate ions coordinate with metal precursors, and the complexes exist in the form of “Bi(CH_3_COO)_3_” and “CH_3_COO^−^‐VO_2_^+^”. The coordination interaction between PEI and Bi(CH_3_COO)_3_ demonstrates the binding energy of −1.49 eV, and the interaction between PEI and CH_3_COO^−^‐VO_2_^+^ by hydrogen bonds with the binding energy of −0.71 eV, corresponding to the binding energy difference of 0.78 eV. However, without the introduction of HOAc, the alkaline conditions provided by PEI enable the metal precursors to exist in the form of “Bi(OH)_3_” and “VO_4_^3−^”. The Bi(OH)_3_ coordinates with PEI, and VO_4_^3−^ interacts with PEI through hydrogen bonds, corresponding to binding energies of −1.28 and −5.61 eV, respectively, with a binding energy difference between the two metal precursors of 4.33 eV. The apparent decrease of binding energy difference from 4.33 to 0.78 eV indicates that the interactions between PEI and different metal precursors are well‐regulated and matched by acetate ions after the introduction of HOAc, which promotes the synthesis of stoichiometric mesoporous BiVO_4_ and confirms the role of “modulator” for acetate ions. Notably, the binding energy between PEI and V^5+^ precursors is significantly reduced from −5.61 to −0.71 eV, while the binding energy between PEI and Bi^3+^ precursors is almost close from −1.28 to −1.49 eV after the introduction of HOAc, demonstrating that the modulation effect of acetate ions can also significantly weaken the interaction of PEI on metal precursors, avoiding the influence of PEI on the original single‐crystalline growth orientation during the synthesis process of metal oligomers. Thus, the selected area electron diffraction (SAED) images of BiVO_4_ samples synthesized with different PEI amounts in the presence of acetate ions show discrete diffraction spots (inset of Figure S5), indicating their single‐crystal structure, which confirms the results obtained by the above theoretical calculations.

The role of PEI in the inorganic–organic composite was further verified by scanning electron microscopy (SEM) images (Figure S5) and N_2_ adsorption–desorption measurements (Figure S6 and Table S2) for the BiVO_4_ samples with different PEI (M_w_ 1800) amounts. As the amount of PEI increased (from 0 to 2.1 g), the morphology of the samples gradually changed (from smooth blocks to rough spheres and then to the mesoporous structure), and the specific surface area and pore volume also gradually increased (from 3 to 31 m^2^/g and from 0.007 to 0.326 cm^3^/g), confirming the “porogenic agent” role of PEI. By subsequent hydrothermal process, the hydrolysis and condensation of metal precursors, along with cross‐linking interaction, enable further improvement of metal oligomers. Furthermore, the dendritic PEIs are interlaced with each other to form a 3D connected spatial network, thereby being well intercalated in the interstitial space of metal oligomers to promote the formation of a mesoporous structure. Finally, after the washing and calcination process, PEI and acetate ions are removed, and metal oligomers are fully crystallized to form MSC BiVO_4_.

**FIGURE 2:**
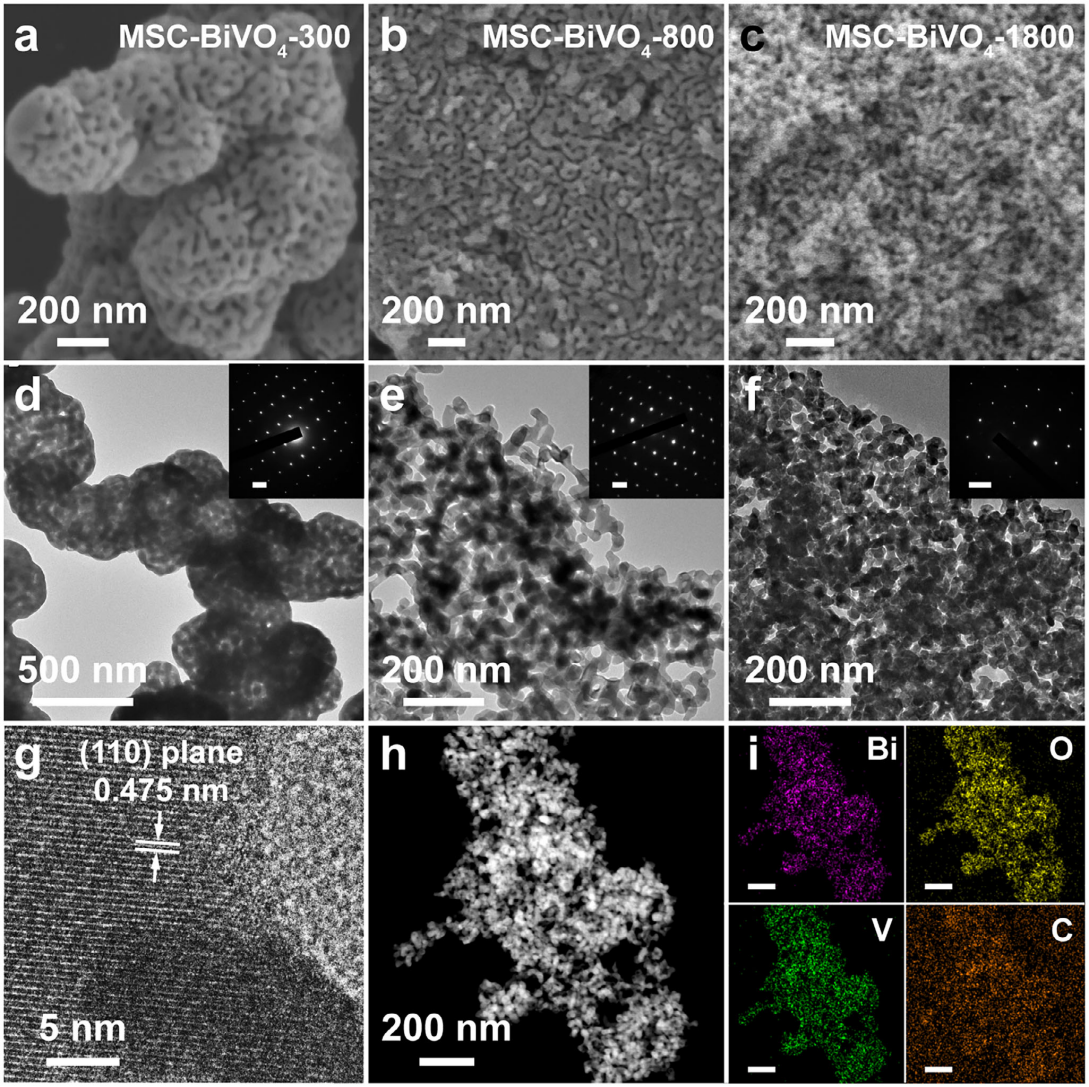
(a–c) SEM images, (d–f) TEM images and (inset of d–f) SAED images of MSC‐BiVO_4_‐300, MSC‐BiVO_4_‐800 and MSC‐BiVO_4_‐1800, respectively; (g) HRTEM image, (h) STEM image and corresponding (i) EDS mapping images of MSC‐BiVO_4_‐1800. (Scale bar: 2 1/nm for the inset of d–f; 200 nm for the element mapping images of (i)).

Moreover, the molecular weight of PEI has a significant impact on the structure of MSC BiVO_4_. The SEM images show the diverse mesoporous structure for the MSC‐BiVO_4_‐300, MSC‐BiVO_4_‐800, MSC‐BiVO_4_‐1800, and MSC‐BiVO_4_‐70000 samples synthesized with various molecular weights of PEI (M_w_ 300, 800, 1800, and 70000) in Figure 2a–c and Figures S7a and S8. Specifically, in the case of small PEI molecular weight (Mw≤ 800), the short‐chain PEI present in the interstices of metal oligomers to form the worm‐like mesopores within the 3D skeleton of BiVO_4_ with the pore wall thickness of 40–50 nm for MSC‐BiVO_4_‐300 (Figure 2a) and MSC‐BiVO_4_‐800 (Figure 2b). With the lengthening of PEI chains (increasing molecular weight to 1800), the PEI chains with suitable length generate a densely cross‐linked mesh structure, which achieves sufficient barrier and intercalation effect for metal oligomers to obtain a mesoporous structure consisting of interconnected branched single crystal BiVO_4_ with the nanoparticle size of 20–30 nm (Figure 2c). While the overlong chain of PEI (molecular weight reaches 70 000) causes large spatial resistance and form the loosely 3D mesh structure, leading to the weakening of the intercalation effect on metal oligomers and the formation of a mesoporous structure stacked by relatively large nanoparticles with thicker walls (50–70 nm) and similar pore structure. The corresponding transmission electron microscopy (TEM) images (Figure 2d–f; Figure S7b) verify the aforesaid pore structure and variations. Based on the above research, the molecular weight of PEI has little impact on the essential properties of PEI, and only changes the pore structure and pore wall thickness by regulating the crosslinking degree of dendritic PEI to modulate the intercalation effect on the growth and crystallization process of metal oligomers without the change of pore size (approximately 30 nm), and thus a series of MSC BiVO_4_ with tunable mesoporous structures and pore wall thicknesses were synthesized.

**FIGURE 3:**
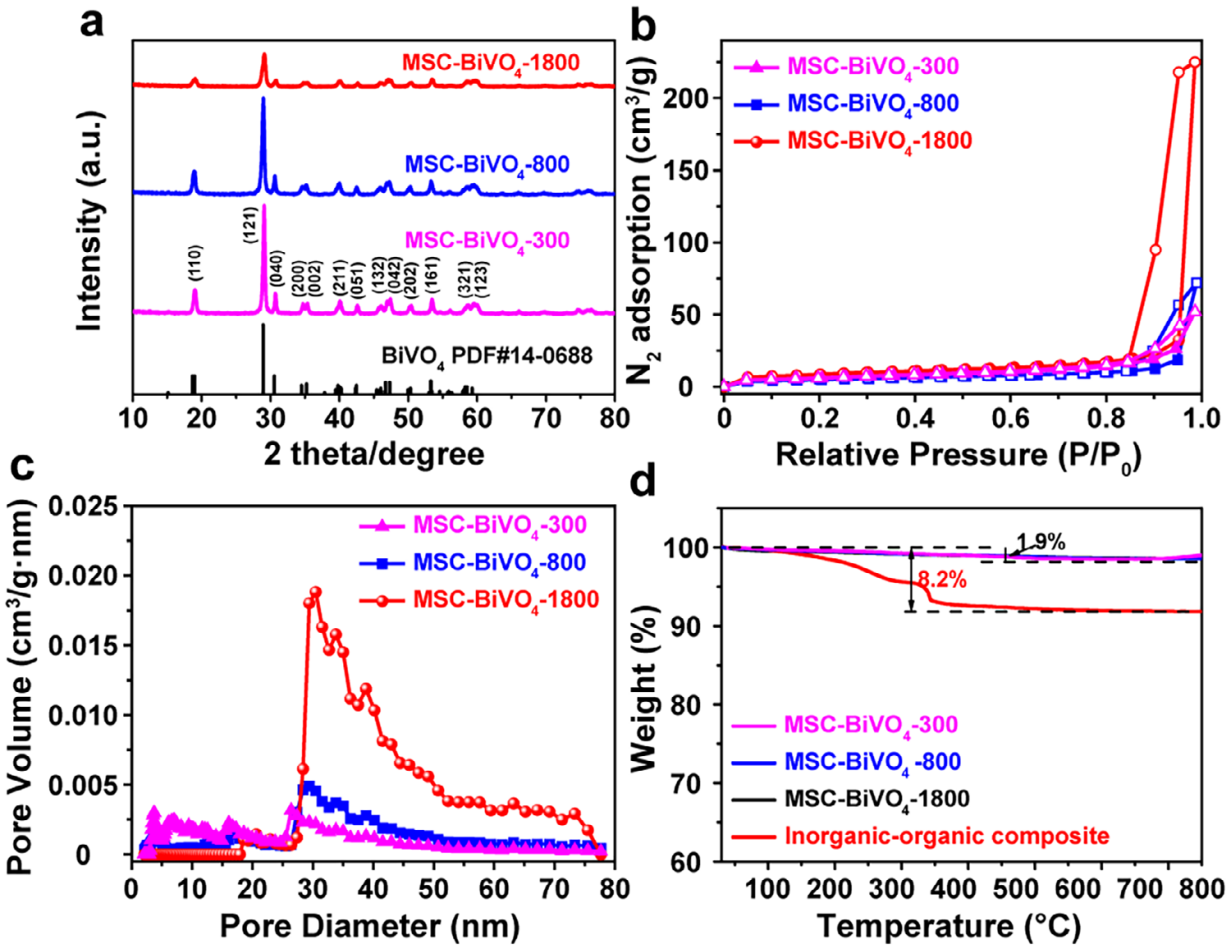
(a) XRD patterns, (b) N_2_ adsorption–desorption isotherms, and (c) corresponding pore size distribution curves of MSC‐BiVO_4_‐300, MSC‐BiVO_4_‐800, and MSC‐BiVO_4_‐1800; (d) TG curves of MSC‐BiVO_4_‐300, MSC‐BiVO_4_‐800, MSC‐BiVO_4_‐1800, and the inorganic–organic composite.

Furthermore, the SAED images (inset of Figure 2d–f; Figure S7c) confirm the single‐crystalline properties of the samples by the apparent discrete diffraction spots. The high‐resolution TEM (HRTEM) of MSC‐BiVO_4_‐1800 (Figure 2g) shows the lattice fringes with a d‐spacing of 0.475 nm, corresponding to the (110) plane of monoclinic BiVO_4_. The scanning transmission electron microscopy (STEM) and corresponding energy dispersive spectroscopy (EDS) element mapping images (Figure 2h,i) display the well‐distribution of Bi, O, and V elements within the MSC‐BiVO_4_‐1800 framework. X‐ray diffraction (XRD) patterns of the samples (Figure 3a; Figure S9a) can be well attributed to the (110), (121), (040), (200), (002), (211), (051), (132), (042), (202), (161), (321) and (123) planes of monoclinic BiVO_4_ (PDF#14‐0688). In addition, Raman spectra of the samples (Figure S10) indicate identical peaks that are indexed to the vibrations of VO_4_3^−^ and V─O bonds, which further verifies the crystal structure of the samples [26, 27]. The N_2_ adsorption–desorption isotherms and corresponding pore size distribution curves were obtained to demonstrate the pore structure of the samples. Figure 3b and Figure S9b display that the N_2_ sorption isotherms of the samples are indexed to the typical type IV curves with H_3_ hysteresis loop at *P/P_0_* = 0.8–1.0, manifesting the presence of large mesopores. The pore size distribution curves (Figure 3c; Figure S9c) obtained by the non‐local density functional theory (NLDFT) pore size distribution model show a similar pore size centered at 29 nm for the samples, which is consistent with the observation results of SEM and TEM images. The specific surface areas of the samples were calculated by using the Brunauer–Emmett–Teller (BET) method and ranged from 18 to 31 m^2^/g for the synthesized mesoporous BiVO_4_ samples (Table S3). The thermogravimetric (TG) analysis (Figure 3d; Figure S9d) displays the weight loss of 8.2% for the inorganic–organic composite and within 2% for the samples after the calcination process, which suggests the introduction of PEI in the inorganic–organic composite and almost no PEI remaining in the BiVO_4_ samples. In addition, the carbon content within 0.4% obtained by CHN analysis (Table S4) for the samples after the calcination process further indicating trace amount of carbon remains. Besides, X‐ray photoelectron spectroscopy (XPS) measurements were conducted to demonstrate the chemical state and elemental composition of the samples. XPS survey spectra (Figure s11a) ascertain that the elemental composition includes Bi, V, O, and C elements. And the XPS Bi 4f and V 2p spectra (Figure S11b,c) manifest the presence of Bi^3+^ and V^5+^, respectively.

**FIGURE 4:**
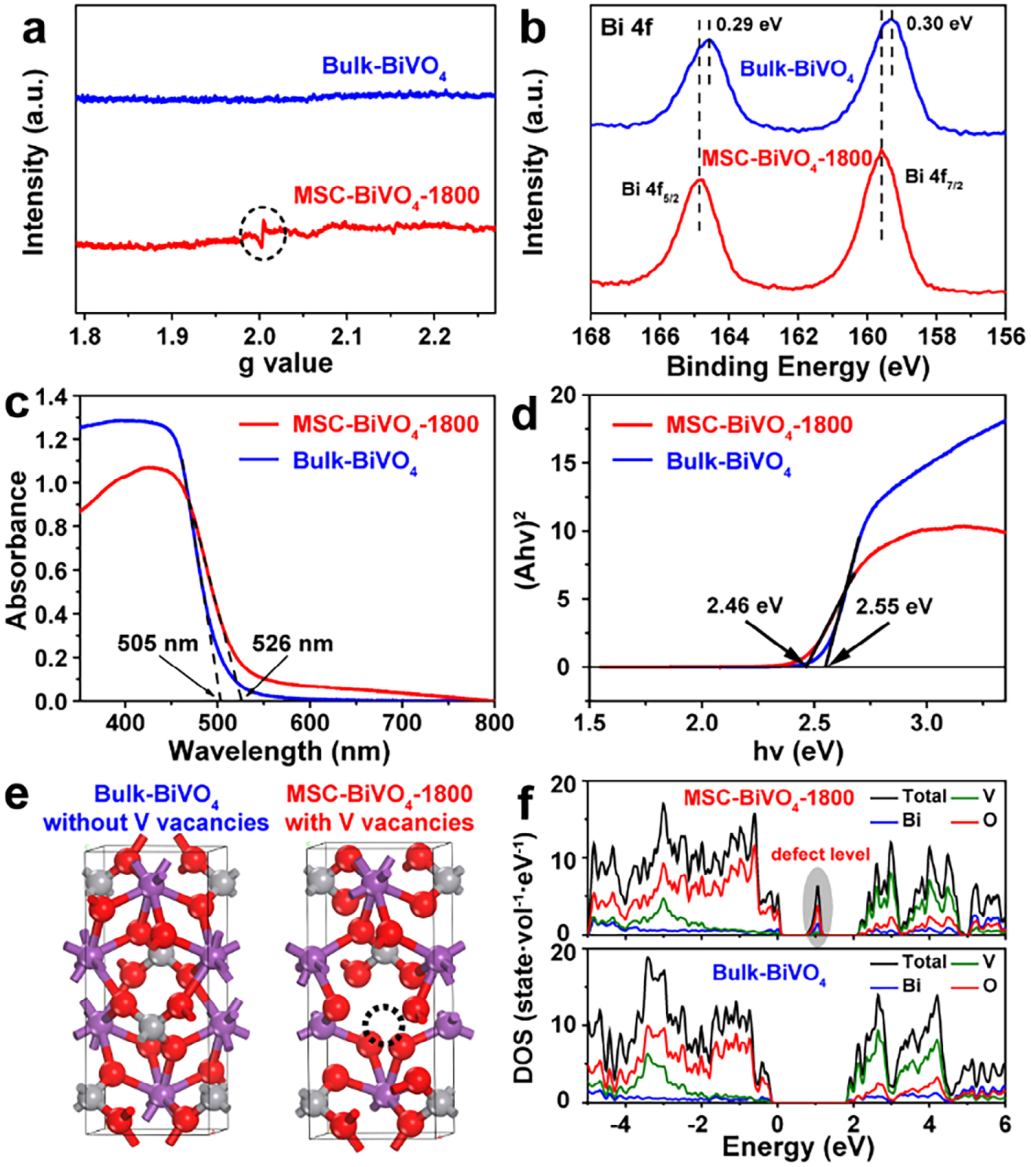
The characterizations and theoretical calculations of MSC‐BiVO_4_‐1800 and Bulk‐BiVO_4_: (a) EPR spectra, (b) XPS Bi 4f spectra, (c) UV–vis diffuse reflection spectra, (d) Plot of (αhv)^2^ vs. hv for estimating the bandgap energy, (e) Crystal structure and (f) corresponding calculated projected density of states.

Owing to the abundant mesopores, high specific surface area, and single‐crystalline structure, the MSC BiVO_4_ materials are considered an ideal material for photocatalytic reaction. Furthermore, the 3D connected mesoporous structure not only facilitates the exposure of adsorption and active sites, but also helps to regulate and construct a microenvironment rich in defects and vacancies. Taking the MSC‐BiVO_4_‐1800 photocatalyst as a typical representative, in order to verify the successful construction of defect sites, a series of characterizations was carried out. The commercial single‐crystalline BiVO_4_ with a nonporous structure and bulk morphology was used as a control sample, named as Bulk‐BiVO_4_ (Figure S14 and Table S5). It is noteworthy that Table S5 shows the lower n_V_:n_Bi_ ratio of MSC‐BiVO_4_‐1800 (0.84 measured by inductive coupled plasma (ICP) spectra and 0.76 obtained by X‐ray fluorescence (XRF) spectra) than Bulk‐BiVO_4_ (0.95 for ICP and 0.81 for XRF), demonstrating the possible existence of V vacancies. Moreover, the blurry lattice fringes in the area circled in Figure S15 also indicate the presence of abundant defects in MSC‐BiVO_4_‐1800 [26, 28, 29, 30]. An apparent electron paramagnetic resonance (EPR) signal was detected for MSC‐BiVO_4_‐1800, while no signal was shown in Bulk‐BiVO_4_ (Figure 4a), which further confirms the existence of abundant V vacancies in MSC‐BiVO_4_‐1800 [28, 31, 32, 33]. XPS V 2p spectra (Figure S16) show identical binding energy of V 2p_3/2_ and V 2p_1/2_ peaks for both photocatalysts. Whereas, Bi 4f of XPS spectra (Figure 4b) display that the Bi 4f_7/2_ and Bi 4f_5/2_ peaks of MSC‐BiVO_4_‐1800 shift to higher binding energy compared to that of Bulk‐BiVO_4_, indicating the elevation of Bi valence for MSC‐BiVO_4_‐1800 to compensate for the missing charges of V^5+^, thereby further proving the formation of V vacancies [32, 34].

For the purpose of further exploring and determining the influence of the V vacancy microenvironment on the bandgap structure, electronic structure, and photocatalytic performance, the UV–vis diffuse reflection spectra and DFT theoretical calculations were conducted. UV–vis diffuse reflection spectra (Figure 4c) display that the light absorption edge of MSC‐BiVO_4_‐1800 is red‐shifted to 526 nm compared to Bulk‐BiVO_4_ (505 nm), which suggests the enlargement of visible light response range for MSC‐BiVO_4_‐1800 with V vacancies [35]. Likewise, the narrower bandgap of MSC‐BiVO_4_‐1800 compared with Bulk‐BiVO_4_ is verified by the plots of (αhv)^2^ vs. hv (Figure 4d). Figure 4e,f shows the crystal structure for defective and stoichiometric BiVO_4_, and the corresponding calculated projected density of states (PDOS) of Bulk‐BiVO_4_ without V vacancies and MSC‐BiVO_4_‐1800 with V vacancies photocatalysts, respectively. A new defect level clearly appeared within the bandgap in MSC‐BiVO_4_‐1800 of Figure 4f, leading to the narrowing of the bandgap, which is consistent with the above‐mentioned characterizations. Such an electronic structure facilitates the excitation of electrons to the conduction band to improve the photoconversion efficiency and expand the visible light response range, thereby promoting the separation of photogenerated carriers [36]. Therefore, the MSC‐BiVO_4_‐1800 photocatalyst with a V vacancy microenvironment can be constructed by the novel and facile synthetic strategy, which is conducive to enhancing its performance in photocatalytic reactions.

**FIGURE 5:**
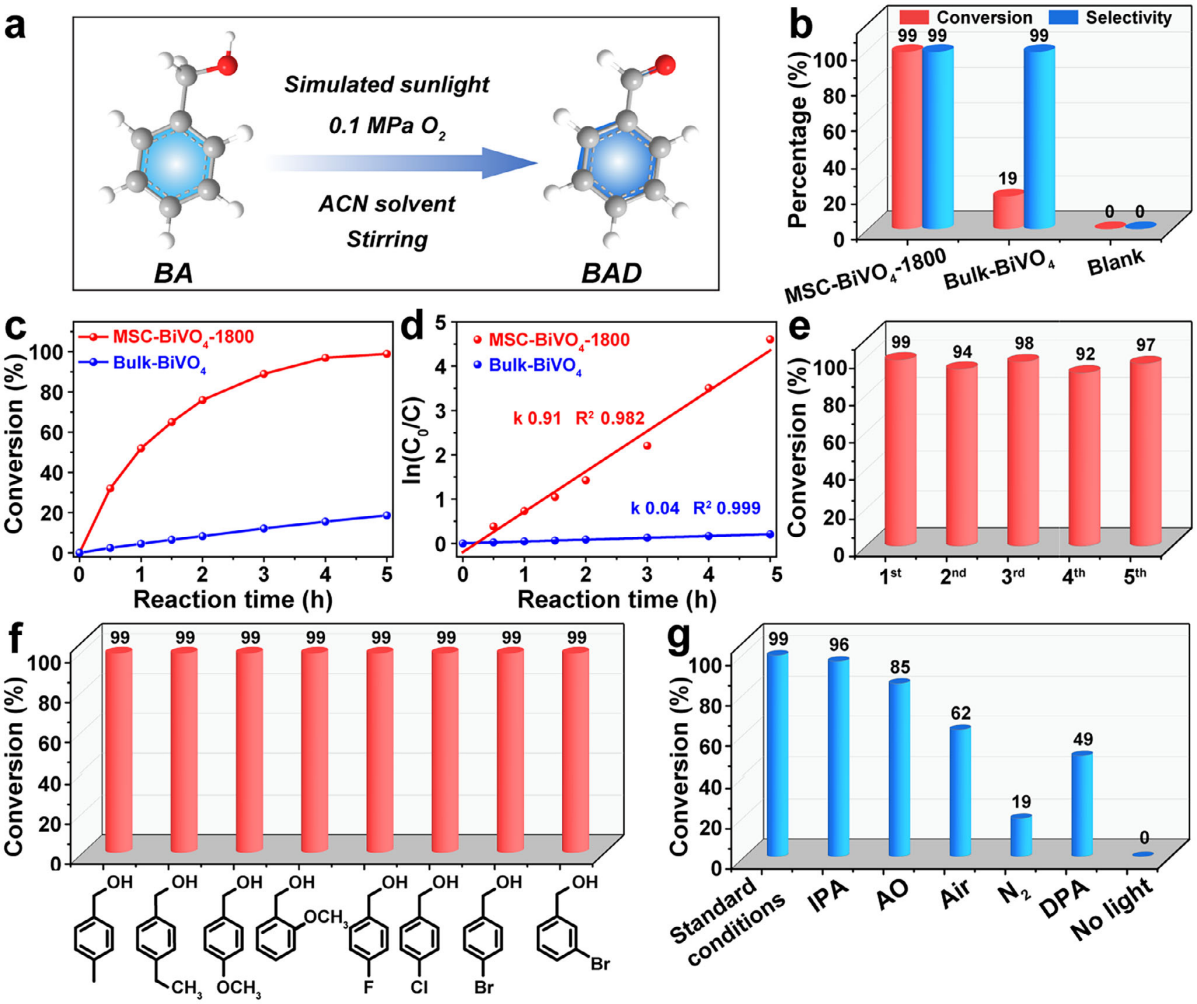
(a) The schematic illustration of photocatalytic selective oxidation for BA to BAD; (b) Photocatalytic performance (conversion and selectivity) of the photocatalysts after 5 h irradiation; (c) Plot of conversion rate vs. reaction time for the photocatalysts; (d) Pseudo‐first‐order kinetics of photocatalytic oxidation of BA for the photocatalysts; (e) 5 times cycling stability test on MSC‐BiVO_4_‐1800 photocatalyst; (f) Photocatalytic conversion of MSC‐BiVO_4_‐1800 for various aromatic alcohols within 5 h; (g) The trapping experiments of conversion rate on MSC‐BiVO_4_‐1800 with different scavengers. (Photocatalytic reaction conditions: 30 mg photocatalyst, 16.7 mM substrate, 3 mL ACN solvent, 1 atm O_2_, Xe lamp with the wavelength range of 350–780 nm acts as simulated sunlight irradiation for 5 h).

The photocatalytic selective oxidation of aromatic alcohols to produce aromatic aldehydes, as an efficient and green synthesis pathway, is of great significance for the preparation of basic compounds in the fine chemical industry [37, 38, 39, 40, 41, 42, 43, 44, 45]. The heterogeneous photocatalytic selective oxidation of BA to benzaldehyde (BAD) was used as an example to investigate the photocatalytic performance of MSC‐BiVO_4_‐1800. Figure 5a displays the schematic illustration of the reaction conditions and process, where the photocatalyst sample is dispersed in acetonitrile (ACN) solvent and the photocatalytic reaction is carried out under an O_2_ atmosphere with stirring. Obviously, MSC‐BiVO_4_‐1800 exhibits excellent catalytic performance with 99% conversion and 99% selectivity under simulated sunlight irradiation for 5 h, which shows a much higher conversion than that of the nonporous Bulk‐BiVO_4_ (19%) with the same selectivity of 99% (Figure 5b). Both photocatalysts display the conversion proportional to the light irradiation time, while the conversion rate of MSC‐BiVO_4_‐1800 increases at a slower rate with increasing light duration, probably owing to the significantly reduced concentration of BA (Figure 5c). The corresponding pseudo‐first‐order kinetics show that the photocatalytic rate constant (*k*) of MSC‐BiVO_4_‐1800 reached 0.91 h^−1^, which was 22.8 times higher than that of Bulk‐BiVO_4_ (0.04 h^−1^), further demonstrating its excellent catalytic performance (Figure 5d). Moreover, MSC‐BiVO_4_‐1800 demonstrates superior catalytic conversion than MSC‐BiVO_4_‐300, MSC‐BiVO_4_‐800, and MSC‐BiVO_4_‐70000 samples (Figure S20), probably due to its higher specific surface area and larger pore volume (Table S3). Almost no BAD was generated (0% conversion) without the introduction of photocatalyst or without simulated sunlight irradiation, which proves that the presence of photocatalyst and light irradiation is necessary for the heterogeneous photocatalytic reaction (Figure 5b,g).

The stability of the photocatalyst is also an important factor for evaluating the catalytic performance, and MSC‐BiVO_4_‐1800 exhibits stable cycling performance with slight decay (within 7%) of conversion rate after 5 times photocatalytic selective oxidation tests (Figure 5e). And the mesoporous structure, crystal structure, and surface chemical state did not change after the cycling tests (Figure S21). The photocatalytic selective oxidation performance of MSC‐BiVO_4_‐1800 for different aromatic alcohols (including p‐methylbenzyl alcohol, p‐fluorobenzyl alcohol, and so on) was further investigated, and the conversion and selectivity were tested to be 99%, suggesting great general adaptability for various aromatic alcohols (Figure 5f; Table S7). To directly identify the primary reactive species involved in the photocatalytic oxidation process for MSC‐BiVO_4_‐1800, a series of trapping experiments was conducted (Figure 5g). Isopropyl alcohol (IPA) and ammonium oxalate (AO) were employed as effective scavengers for hydroxyl radicals (•OH) and h^+^, respectively. IPA rapidly reacts with •OH via hydrogen abstraction, while AO irreversibly consumes h^+^ through an oxidative decarboxylation pathway. The role of superoxide radicals (•O_2_^−^) was investigated by comparing reaction performance under air and inert N_2_ atmospheres, a decisive approach since molecular oxygen serves as the essential precursor for •O_2_^−^ formation. Furthermore, diphenylamine (DPA) served as a specific probe for oxygen‐centered radical intermediates generated during the oxidation pathway of BA. The oxidation of DPA to its stable nitroxide derivative via hydrogen atom transfer by the oxygen‐centered radicals provides direct evidence for the existence of such radical intermediates. The pronounced inhibition of BA conversion rate upon the introduction of these scavengers or under air and N_2_ atmosphere provides convincing evidence for confirming the vital active species and intermediates in the proposed reaction mechanism. The introduction of IPA did not affect the conversion, which indicates that •OH radicals were hardly generated during the photocatalytic reaction. This is presumably due to the utilization of acetonitrile‐based rather than aqueous solvent systems, which inherently limit the formation of •OH. The conversion decreased to 85%, 62%, and 19% with the introduction of AO, and under air and N_2_ conditions, respectively, verifying that h^+^ and •O_2_^−^ play a vital role in the heterogeneous photocatalytic oxidation process. Moreover, the *quasi* in situ electron spin resonance (ESR) experiments were further performed to verify the vital active species in the photocatalytic reaction system. As shown in Figure S22a, the triplet ESR signals observed for MSC‐BiVO_4_‐1800 under dark conditions can be attributed to 2,2,6,6‐tetramethylpiperidin‐1‐oxyl (TEMPO). After simulated visible‐light irradiation, the intensity of these triplet signals significantly decreased. This indicates that the photogenerated h^+^ reacted with TEMPO, leading to its oxidation and confirming the substantial generation of the key active species of photogenerated h^+^ [46, 47]. In Figure S22b, 5,5‐dimethyl‐1‐pyrroline N‐oxide (DMPO) was used to capture reactive oxygen species. No signals were detected under dark conditions, but after irradiation for 10 min, characteristic signals attributed to •O_2_^−^ obviously appeared, demonstrating the efficient formation of another key active species, •O_2_^−^. In addition, the oxygen‐centered radicals were verified to be the predominant intermediates by the decrease of photocatalytic conversion from 99% to 49% after the introduction of DPA [48].

**FIGURE 6:**
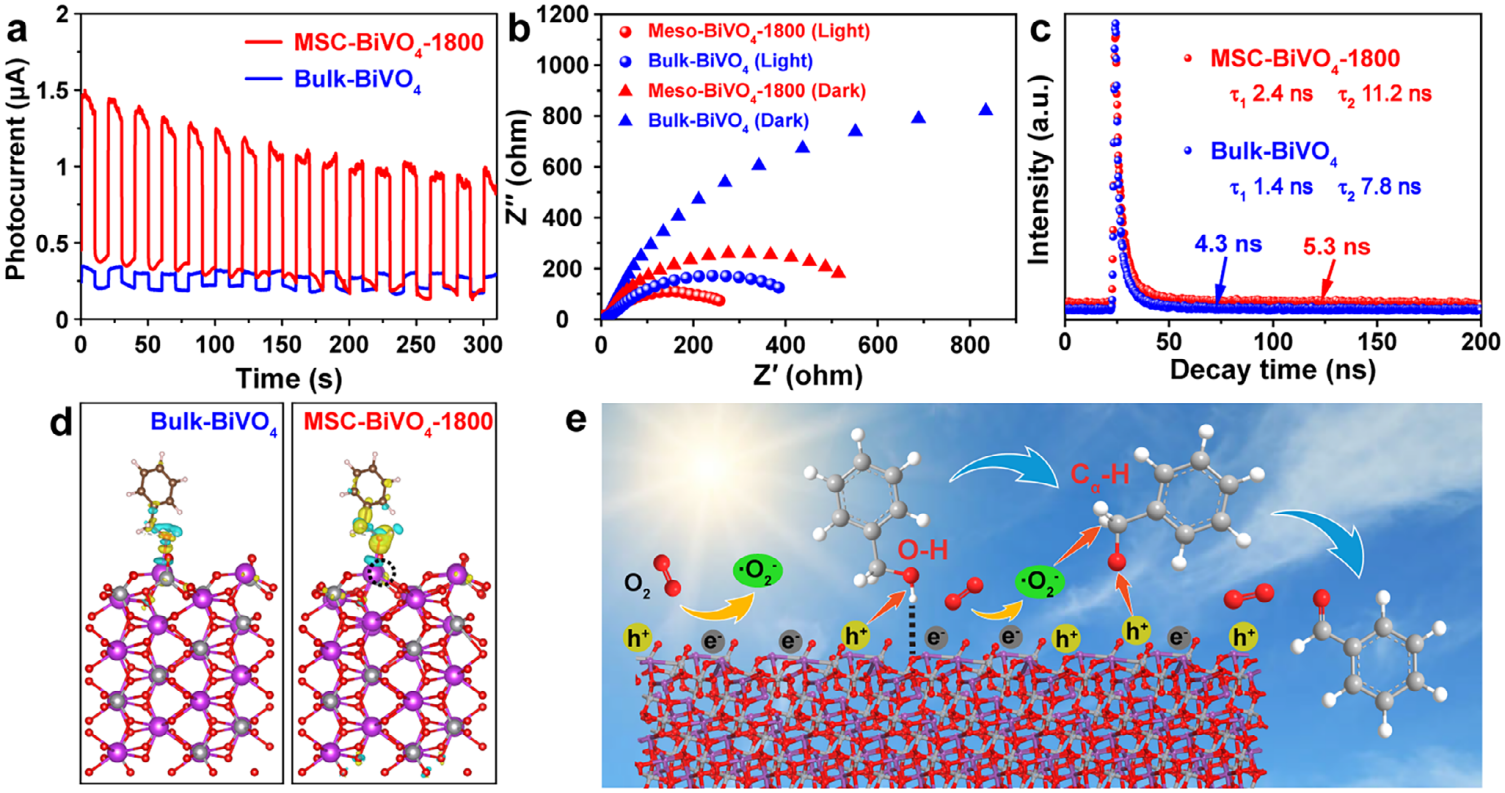
The characterizations and theoretical calculations of MSC‐BiVO_4_‐1800 and Bulk‐BiVO_4_: (a) Transient photocurrent responses, (b) EIS Nyquist plots, (c) TRPL spectra, (d) The charge difference density calculations of a BA molecule adsorbed on (110) plane, (e) The proposed possible pathway for photocatalytic selective oxidation of BA on MSC‐BiVO_4_‐1800.

The photoelectrochemical and optical characterizations—including transient photocurrent response, electrochemical impedance spectroscopy (EIS) Nyquist plots with and without light irradiation, and photoluminescence (PL) spectra (Figure 6a,b; Figure S23)—collectively demonstrate the superior charge carrier separation in MSC‐BiVO_4_‐1800. Specifically, its enhanced and stable photocurrent response reflects more efficient charge carrier separation and utilization; the smaller Nyquist semicircle diameter in the EIS plot indicates lower charge transfer resistance; and its reduced PL emission intensity further confirms suppressed charge carrier recombination [49, 50]. Besides, time‐resolved photoluminescence (TRPL) emission decay spectra (Figure 6c) were conducted to measure the carrier lifetime of MSC‐BiVO_4_‐1800 and Bulk‐BiVO_4_ with the average lifetime of 5.3 and 4.3 ns, respectively (Table S8) [51, 52, 53]. Collectively, these characterizations demonstrate that the V vacancy microenvironment promotes the separation of photogenerated electron–hole pairs. This enhancement primarily stems from its optimized surface electronic structure—as predicted by the PDOS analysis in Figure 4f—which promotes interfacial charge transfer kinetics. The rapid scavenging of separated charge carriers through interfacial reactions significantly inhibits the recombination of photogenerated electron–hole pairs [36]. Moreover, the charge density difference calculations were performed to reveal the electron redistribution behavior for a BA molecule adsorbed on the (110) plane of both photocatalysts. The presence of a V vacancy microenvironment introduces localized electronic states within the bandgap of BiVO_4_ (verified by PDOS analysis in Figure 4f), which effectively increases the density of available electrons near the Fermi level. This significantly lowers the energy barrier for electron transfer, making it more favorable for electrons to migrate from the photocatalyst surface to the adsorbed BA molecule. Consequently, as shown in Figure 6d, the V vacancies can induce more electrons transfer to O and C_α_ atoms of the BA molecule, which accelerates the breaking of the ─OH and C_α_─H bonds, thus enhancing the activation of BA and promoting the heterogeneous photocatalytic oxidation reaction [54, 55].

Consequently, the proposed photocatalytic oxidation mechanism was illustrated in Figure 6e. Under simulated sunlight irradiation, photogenerated electron–hole pairs are rapidly generated, and photogenerated electrons activate molecular oxygen to generate •O_2_^−^. The h^+^ and •O_2_^−^ act as reactive species to abstract a hydrogen atom (H) from the Cα─H bond of the BA molecule adsorbed on the catalyst surface, generating a neutral benzyl radical intermediate (C_6_H_5_CH_2_O•). This benzyl radical was subsequently attacked to form oxygen‐centered radicals, and the target product BAD was formed after further dehydrogenation of H• from the C_α_─H bonds. During the photocatalytic oxidation process, the single‐crystalline properties without a grain boundary can efficiently inhibit the recombination of charge carriers and boost their transfer efficiency. The abundant mesoporous channels are beneficial to expose more adsorption sites for the BA molecule and accelerate mass transfer of BA and BAD molecules. Moreover, the 3D connected mesoporous structure is also conducive to the fabrication of a V vacancy microenvironment that not only narrows the bandgap width and broadens the visible light absorption range, but also enhances the activation of BA and facilitates the separation of photogenerated electron–hole pairs, thereby exhibiting excellent photocatalytic performance for MSC‐BiVO_4_‐1800.

## 3 Conclusions

In conclusion, a polymer‐intercalated modulation assembly strategy is developed to synthesize MSC BiVO_4_ with a tunable pore structure. In this strategy, the co‐assembly of metal precursors, acetate ions, and PEI was achieved by the coordination bonds and hydrogen bonds. The dendritic PEIs act as a porogenic agent to form an interconnected 3D spatial network and are fully intercalated into metal oligomers to promote the formation of mesoporous structures. Furthermore, the “modulator” acetate ions can match the interactions between the two metal precursors and PEI, and weaken the influence of PEI on the original single‐crystalline growth orientation of metal oligomers, thereby realizing the synthesis of MSC BiVO_4_. The as‐prepared MSC‐BiVO_4_‐1800 photocatalyst exhibits superior performance for the selective oxidation of various aromatic alcohols, which is due to the single‐crystalline properties and 3D connected mesoporous structure with a V vacancy microenvironment. This study provides a unique idea and strategy to realize the controllable synthesis of mesoporous single‐crystalline materials and construct high‐performance photocatalysts, which is of great significance for the future development of photocatalysis.

## 4 Experimental Section

### 4.1 Synthesis of Mesoporous BiVO_4_‐1800 (MSC‐BiVO_4_‐1800)

A total of 2.1 g PEI (M_w_ 1800) was dissolved in 30 mL of deionized water to obtain a transparent solution. The 70 mmol of acetic acid (HOAc), 0.5 mmol of Bi(NO_3_)_3_·5H_2_O, and 0.4 mmol of NH_4_VO_3_ were added to the above solution in turn with 30 min interval under stirring conditions. Subsequently, the mixed solution was stirred for 30 min and transferred to a 100 mL Teflon‐lined stainless‐steel autoclave, and heated at 100°C for 24 h. The obtained powder was washed with deionized water and anhydrous ethanol, dried at 70°C for 12 h, calcinated at 350°C for 3 h, and named as MSC‐BiVO_4_‐1800.

### 4.2 Synthesis of Mesoporous BiVO_4_‐300, Mesoporous BiVO_4_‐800 and Mesoporous BiVO_4_‐70000 (MSC‐BiVO_4_‐300, MSC‐BiVO_4_‐800 and MSC‐BiVO_4_‐70000)

A total of 3.5 g PEI (M_w_ 300), 4.8 g PEI (M_w_ 800), and 2.5 g PEI (M_w_ 70000) were dissolved in 30 mL of deionized water, respectively. The 70 mmol of HOAc, 0.5 mmol of Bi(NO_3_)_3_·5H_2_O, and 0.4 mmol of NH_4_VO_3_ were added to the above solutions in turn with 30 min interval under stirring conditions. Subsequently, both mixed solutions were stirred for 30 min and transferred to a 100 mL Teflon‐lined stainless‐steel autoclave, and heated at 100°C for 24 h. The obtained samples were washed with deionized water and anhydrous ethanol, dried at 70°C for 12 h, calcinated at 350°C for 3 h, and named as MSC‐BiVO_4_‐300, MSC‐BiVO_4_‐800, and MSC‐BiVO_4_‐70000, respectively.

### 4.3 Density Functional Theory (DFT) Calculation Methods

The calculations about the synthesis process were conducted by the Gaussian 16, C01 software package. The hybrid functional PBE0 functional was chosen for all calculations in combination with the D3 version of Grimme's dispersion with Becke–Johnson damping (DFT‐D3BJ). The V/Bi uses the SDD basis set, while the others utilize the 6–31G+(d,p) basis set for geometry optimization and frequency calculations. The single‐point energy calculations were performed by using the sdd4 and 5 basis sets of V/Bi and the other 6–311+G(d,p)4 and 9 basis sets. The interaction energy between PEI and metal precursors was calculated by the following formula E_bind_ = E_complex_‐(E_partA_+E_partB_). Besides, all the DFT calculations for structural and photocatalytic sections were used in the Vienna ab initio simulation (VASP5.4.4) code. The exchange‐correlation is simulated with the PBE functional, and the ion‐electron interactions were described by the PAW method. The vdWs interaction was included by using the empirical DFT‐D3 method. The Monkhorst–Pack‐grid‐mesh‐based Brillouin zone k‐points are set as 3 × 2 × 1 for all periodic structures with the cutoff energy of 400 eV. The convergence criteria are set as 0.01 eV A^−1^ and 10^−4^ eV in force and energy, respectively. At least a 15 Å vacuum layer along the *z* direction is employed to avoid interlayer interference. The pure BiVO_4_(110) surface and the BiVO_4_(110) surface with a V vacancy were used to support the benzyl alcohol rely on the Bi‐O interaction.

### 4.4 Photocatalytic Activity Tests

The photocatalytic aromatic alcohols oxidation reaction was performed in a sealed 50 mL quartz reactor. 30 mg photocatalyst was added to the reactor containing 3 mL acetonitrile and 0.05 mmol aromatic alcohols. Then, the suspension was stirred and infused with O_2_ for 30 min in the dark to ensure adequate adsorption–desorption equilibrium between photocatalyst and aromatic alcohols, and filled with O_2_ gas before photocatalytic reaction. The suspension was irradiated with the simulated sunlight from an Xe arc lamp (350–780 nm). After irradiation for 5 h, an equal volume of the liquid sample was obtained by a syringe to filter the photocatalyst with a 0.22 µm filter and analyzed by an Agilent Gas Chromatograph (GC 7890B). The conversion of aromatic alcohols and the selectivity of aromatic aldehydes were calculated as follows:

(1) $$Conversion\;\left(\%\right)=\left[\left(C_{0}-C_{\mathrm{alcohol}}\right)/C_{0}\right]\times 100\%$$

(2) $$Selectivity\;\left(\%\right)=\left[C_{\mathrm{aldehyde}}/\left(C_{0}-C_{\mathrm{alcohol}}\right)\right]\times 100\%$$

### 4.5 Photoelectrochemical Performance

The photoelectrochemical performance was performed by using a three‐electrode CHI 660E electrochemical workstation with the samples as the working electrodes, a saturated calomel electrode (SCE) as reference electrode, and Pt foil as counter electrode in a 0.5 m Na_2_SO_4_ electrolyte. The working electrodes were prepared by dropping the catalyst suspension consisting of 10 mg powder and 0.2 mL ethanol onto the fluorine‐doped tin oxide glass (0.5 cm × 0.5 cm). And the AM 1.5 solar power system was used as a light source. Specifically, the transient photocurrent responses and electrochemical impedance spectroscopy (EIS) Nyquist plots were conducted at applied bias of 0.4 and 1.4 V vs. SCE, respectively.

## Conflicts of Interest

The authors declare no competing interest.

## Supporting information




**Supporting File**: advs74033‐sup‐0001‐SuppMat.docx.

## Data Availability

The data that support the findings of this study are available from the corresponding author upon reasonable request.
