# Supplementary material for: A Polymer‐Intercalated Modulation Assembly Strategy Towards Mesoporous Single‐Crystalline BiVO4 Materials for Enhanced Photocatalytic Performance
Source: Adv Sci (Weinh). 2026 Jan 27;13(19):e22515. doi: 10.1002/advs.202522515 (PMC13045432; doi:10.1002/advs.202522515)
Supplement: Supplementary file 1 — Supporting File: advs74033‐sup‐0001‐SuppMat.docx. [file ADVS-13-e22515-s001.docx]

Supplementary Information

A Polymer-intercalated Modulation Assembly Strategy towards Mesoporous Single-crystalline BiVO_4_ Materials for Enhanced Photocatalytic Performance

*Wei Li, Xiaoyan, Wei, Yumeng Mao, Zhengwen Tan, Wenjun Duan, Ling Zhang, Zhen-An Qiao**

W. Li, Y. Mao, Z. Tan, Prof. Z.-A. Qiao

Jilin University

State Key Laboratory of Inorganic Synthesis and Preparative Chemistry, College of Chemistry, Qianjin Street 2699, Changchun 130012, P. R. China

*Corresponding Author: qiaozhenan@jlu.edu.cn

W. Li, W. Duan

Shanxi University

Shanxi Key Laboratory of Coal-based Value-added Chemicals Green Catalysis Synthesis, School of Chemistry and Chemical Engineering, Wucheng Road 92, Taiyuan 030006, P. R. China

X. Wei, L. Zhang

Jilin University

State Key Laboratory of Supramolecular Structure and Materials, College of Chemistry, Qianjin Street 2699, Changchun 130012, P. R. China

**Contents:**

**Section 1. Experimental Section**

**Section 2. Supplementary Figures**

**Section 3. Supplementary Tables**

**Section 1. Experimental Section**

1 Materials

All the reagents were used without further purification. Polyethyleneimine (PEI, average M_w_ = 800) was purchased from Sigma-Aldrich Co., Ltd. Glacial acetic acid (C_2_H_4_O_2_, AR) and benzyl alcohol (C_7_H_8_O, AR) were obtained from Sinopharm Chemical Reagent Co., Ltd, China. Polyethyleneimine (PEI, average M_w_ = 1800 and 70000), bismuth nitrate pentahydrate (Bi(NO_3_)_3_·5H_2_O, AR), bismuth vanadate (BiVO_4_, 98%), ammonium oxalate ((NH_4_)_2_C_2_O_4_·H_2_O, AR), diphenylamine (C_12_H_11_N, GC), 4-methylbenzyl alcohol (C_8_H_10_O, 98%), 4-ethylbenzyl alcohol (C_9_H_12_O, GC), 4-methoxybenzyl alcohol (C_8_H_10_O_2_, 98%), 2-methoxybenzyl alcohol (C_8_H_10_O_2_, 98%), 4-fluorobenzyl alcohol (C_7_H_7_FO, GC), 4-chlorobenzyl alcohol (C_7_H_7_ClO, 99%) and 3-bromobenzyl alcohol (C_7_H_7_BrO, 99%) were purchased from Aladdin Industrial Co., Ltd, China. Ammonium metavanadate (NH_4_VO_3_, AR) was purchased from Kermel Chemical Reagent Co., Ltd, China. 4-bromobenzyl alcohol (C_7_H_7_BrO, 99%) was obtained from Energy Chemical Co., Ltd, China. Ethanol absolute (C_2_H_5_OH, AR), acetonitrile (CH_3_CN, AR) and isopropyl alcohol (C_3_H_8_O, AR) were obtained from Tiantai Chemical Co., Ltd, China. The deionized water (18.2 MΩ cm resistivity) was obtained by a PALL PURELAB Plus instrument. The oxygen (O_2_) and nitrogen (N_2_) gas were high purity (99.999%) in the experiment.

2 Characterization

The morphology was measured by a JEOL JSM 6700F Scanning Electron Microscopy (SEM). Transmission electron microscopy (TEM), high-resolution TEM (HRTEM), selected area electron diffraction (SAED), scanning transmission electron microscopy (STEM) and corresponding EDS were performed on a Philips-FEI Tecnai G2S-Twin microscope (200 kV field emission gun). N_2_ adsorption-desorption isotherms were collected from a Quanta 4200e surface area analyzer at -196 °C and the pore size distribution was calculated according to the density functional theory pore size distribution (NLDFT) model. X-ray diffraction (XRD) measurement was conducted on a Bruker D2 Advance X-ray diffractometer by using Cu-Kα radiation (30 kV, 15 mA, *λ* = 0.15418 nm). Fourier transform-infrared spectroscopy (FT-IR) measurements were performed on an IFS-66V/S infrared spectrophotometer. Thermogravimetric (TG) analyses were carried out on a thermogravimetric analyzed instrument (TGA Q500). The measurements were performed from 50 to 800 °C under air atmosphere, with a heating rate of 10 °C·min^-1^. ^13^C Solid-state NMR (cross polarization magic-angle spinning (CP/MAS) spectra were carried out on a Bruker Avance 600 MHz spectrometer. Raman spectra were performed on a Renishaw via Raman microscope. The carbon content of the samples was obtained by a Elementar Vario MACRO cube CHNS analyzer. X-ray fluorescence (XRF) analysis was performed by a PANalytical Epsilon1 X Fluorescence Spectrometer. Inductive coupled plasma emission spectroscopy (ICP) measurements were carried out on an iCAP 7600 ICP-OES spectrometer. X-ray photoelectron spectroscopy (XPS) tests were conducted on an ESCALAB 250 X-ray photoelectron spectrometer by using Al K*α* (1486.8 eV) as the X-ray source. The UV-vis diffuse reflectance spectra were conducted by a Shimadzu U-4100 spectrometer. The *quasi in-situ* electron spin resonance (ESR) measurements (Bruker ESR5000) were performed to explore the vital active species by using 2,2,6,6-tetramethylpiperidin-1-oxyl‌ (TEMPO) and 5,5-dimethylpyridine N-oxide (DMPO) during the photocatalytic process. The photoluminescence (PL) emission spectra were carried out on a luminescence spectrophotometer (FLUOROMAX-4) with a 300 nm filter (280 nm excitation). Time-resolved fluorescence spectra (TRPL) were conducted on a FLS920 luminescence spectrophotometer. X-band electron paramagnetic resonance (EPR) was performed by EMPplus-10/12.

**Section 2. Supplementary Figures**





Figure S1. FT-IR spectra of the sample synthesized without HOAc and its corresponding precursor (*i.e.* without calcination), and PEI.

As shown in FT-IR spectra (Figure S1), the asymmetrical υ^as^(COO^-^) and symmetrical υ^s^(COO^-^) stretching vibrations are disappeared in the precursor prepared without HOAc addition, which also verifies the coordination interaction of metal ions with acetate ions after the introduction of HOAc.





Figure S2. FT-IR spectra of PEI, PEI/HOAc/Bi^3+^/V^5+^ metal oligomers inorganic-organic composite synthesized with different hydrothermal times (1-12 h).

The FT-IR spectra of the inorganic-organic composite synthesized with different hydrothermal times (Figure S2) indicate that the peaks of hydrogen bonds between the metal oligomers and PEI (3438 cm^-1^), and the coordination bonds between the acetate ions and the metal oligomers (1467 and 1384 cm^-1^) stably exist and without peak shift occur during the hydrothermal synthesis process. Besides, the FT-IR peaks ascribed to C-N bonds (1071 cm^-1^), NH groups (1615 cm^-1^) and methylene groups (2855 and 2961 cm^-1^) of PEI also stably exist during the hydrothermal synthesis process. These further verify the formation of PEI/HOAc/Bi^3+^/V^5+^ metal oligomers inorganic-organic composite by hydrogen bonds and coordination bonds.


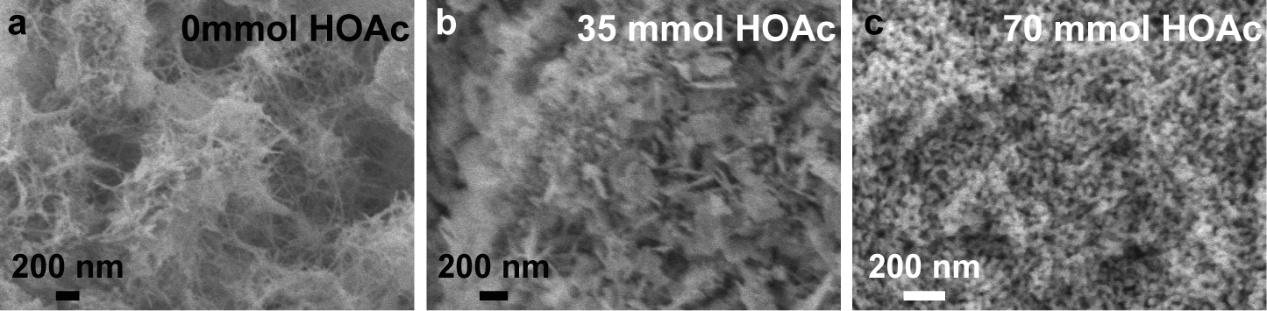


Figure S3. The synthesized samples with different amount of HOAc: (a) 0 mmol HOAc, (b) 35 mmol HOAc, (c) 70 mmol HOAc.





Figure S4. XRD patterns of the samples synthesized with different HOAc amount (0-70 mmol).

For the purpose of exploring the role of acetate ions, we regulate the amount of HOAc (0-70 mmol) during the synthesis process (Figure S3 and Table S1). With the increase of HOAc amount, the morphology of the samples gradually changed from nanowires (Figure S3a) and nanosheets (Figure S3b) to worm-like mesoporous structures (Figure S3c), thus experimentally verifying that the crucial role of acetate ions for the formation of mesoporous structures. Besides, the corresponding crystal phase undergoes significant changes. That is, as the HOAc amount rises, bismuth vanadate gradually changes from Bi_2_VO_5.5_ with non-stoichiometric ratio to BiVO_4_ with stoichiometric ratio (Figure S4). The above demonstrates that the regulation of acetate ion amount exhibits a significant impact on the porous structure and crystal structure of bismuth vanadate materials. Therefore, it confirms that acetate ions play a crucial role during the synthesis of MSC BiVO_4_.

**
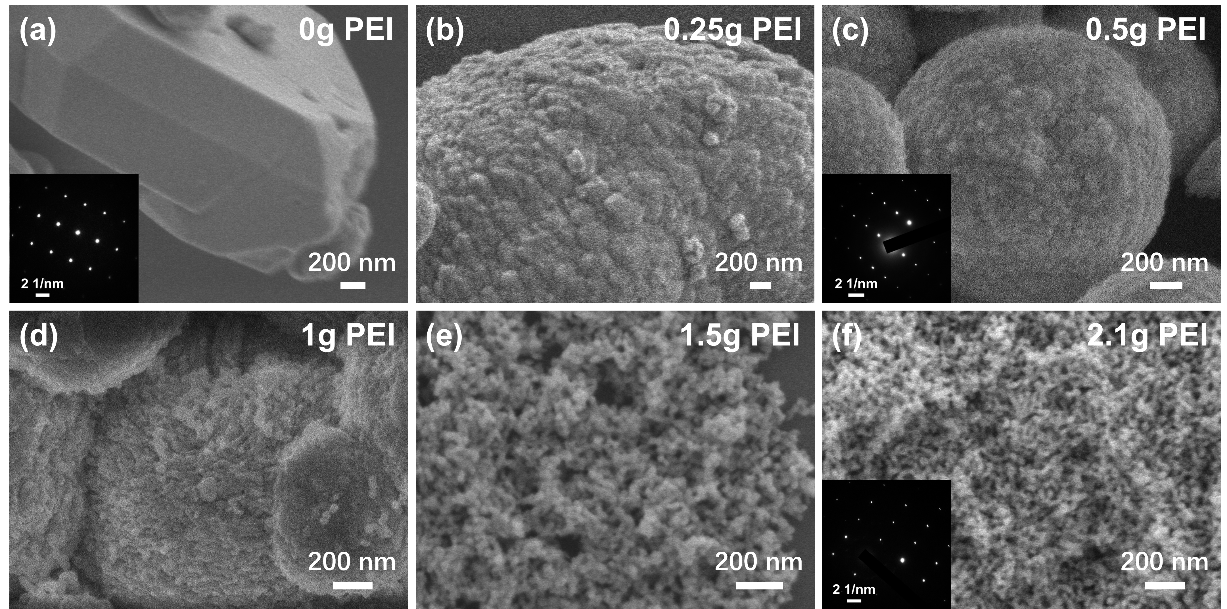
**

Figure S5. SEM images of BiVO_4_ samples synthesized with different PEI amount: (a) 0 g PEI, (b) 0.25 g PEI, (c) 0.5 g PEI, (d) 1.0 g PEI, (e) 1.5 g PEI, (f) 2.1 g PEI.

The pore size shown in Table S1 is apparently expanded from 4 to 29 nm for the samples synthesized with increasing PEI amount, which can also be observed in the SEM images of Figure S5.


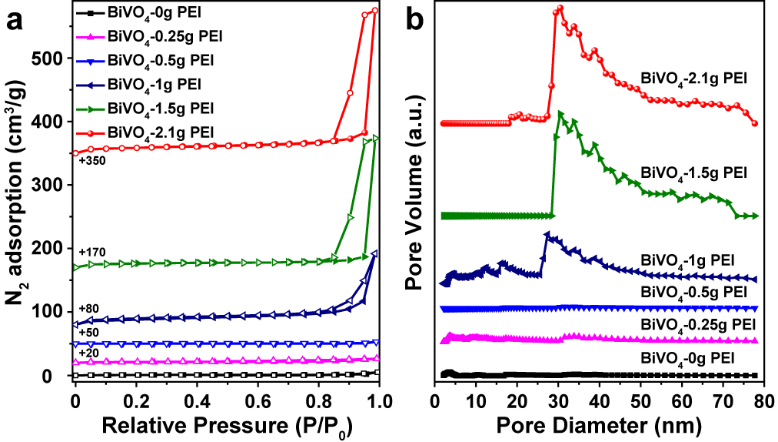


Figure S6. (a) N_2_ adsorption-desorption isotherms (the magenta, blue, navy, olive and red lines offset by 20, 60, 80, 170 and 350 cm^3^/g, STP, respectively, for clarity) and (b) corresponding pore size distribution curves of BiVO_4_ samples synthesized with different PEI amount (0-2.1 g).


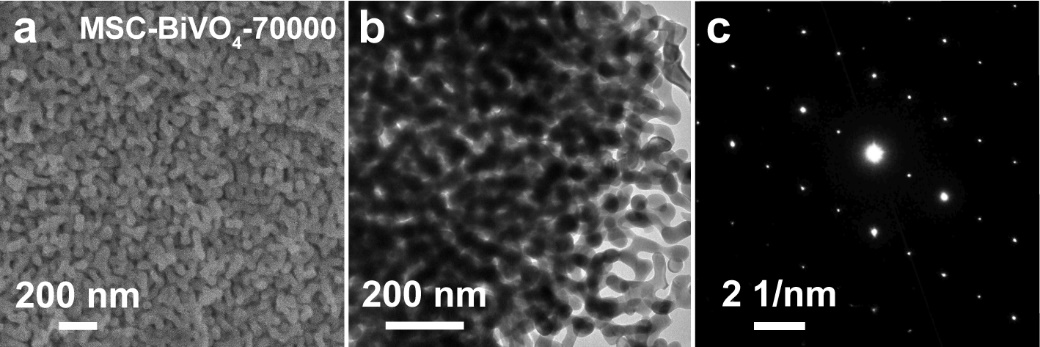


Figure S7. (a) SEM image, (b) TEM image and (c) HRTEM image of MSC-BiVO_4_-70000.

The HRTEM images of MSC-BiVO_4_-70000 (Figure S7c) also demonstrates the single-crystalline properties.

**
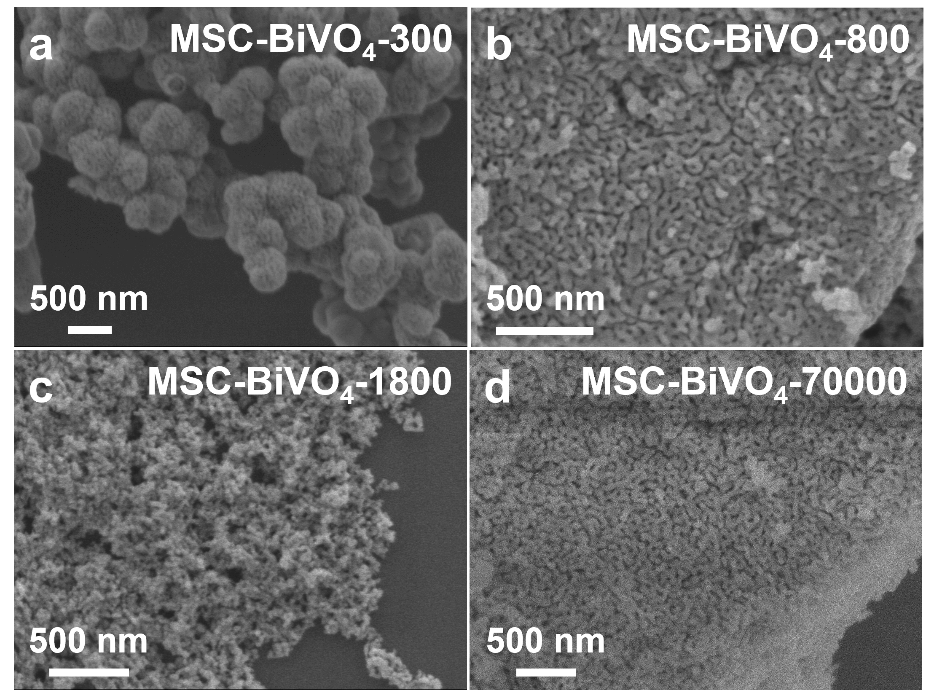
**

Figure S8. Low magnification SEM images of (a) MSC-BiVO_4_-300, (b) MSC-BiVO_4_-800, (c) MSC-BiVO_4_-1800 and (d) MSC-BiVO_4_-70000.

**
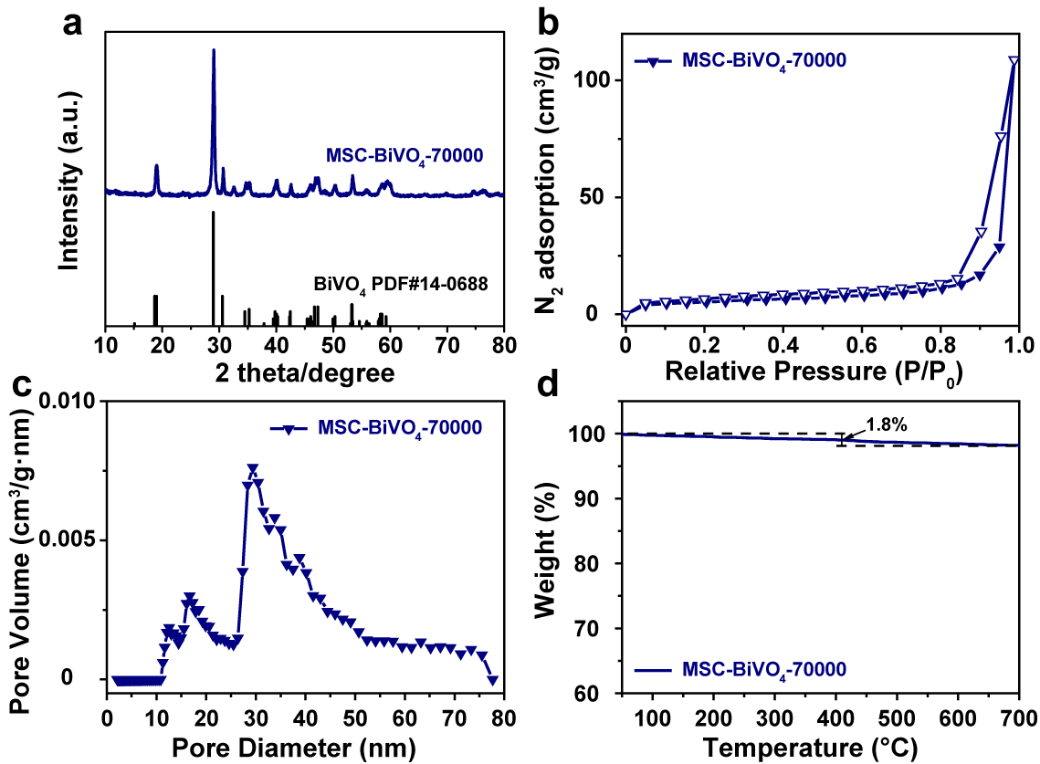
**

Figure S9. (a) XRD patterns, (b) N_2_ adsorption-desorption isotherms and (c) corresponding pore size distribution curves, (d) TG curves of MSC-BiVO_4_-70000.

**

**

Figure S10. Raman spectra of MSC-BiVO_4_-300, MSC-BiVO_4_-800, MSC-BiVO_4_-1800 and MSC-BiVO_4_-70000.

**
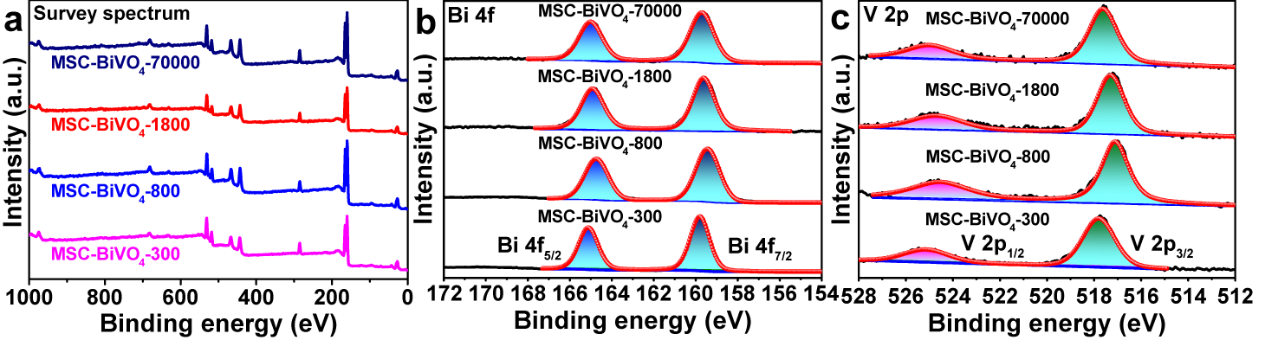
**

Figure S11. XPS spectra of MSC-BiVO_4_-300, MSC-BiVO_4_-800, MSC-BiVO_4_-1800 and MSC-BiVO_4_-70000: (a) Survey spectra, (b) Bi 4f, (c) V 2p.

XPS Bi 4f spectra (Figure S11b) show that the peaks located at around 164.9 eV and 159.6 eV are well ascribed to Bi 4f_5/2_ and Bi 4f_7/2_, respectively, suggesting the existence of Bi^3+^. The peaks of 524.7 eV and 517.3 eV in V 2p spectra (Figure S11c) are identified to V 2p_1/2_ and V 2p_3/2_, respectively, which demonstrates the presence of V^5+^.


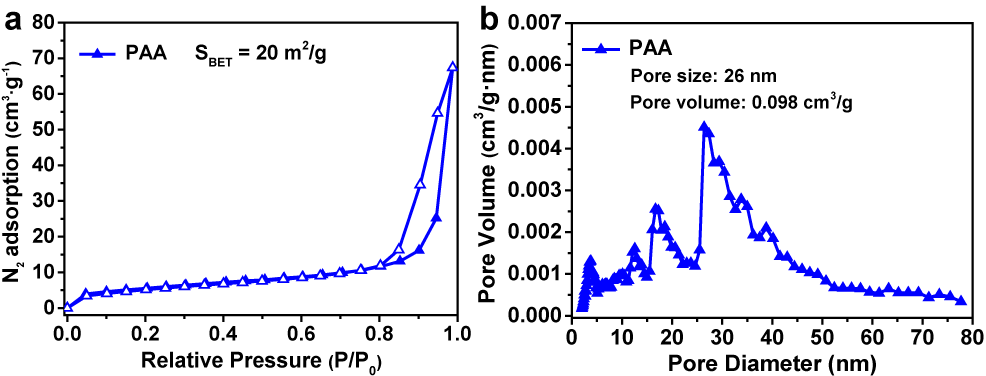


Figure S12. (a) The N_2_ adsorption-desorption isotherms and (b) corresponding pore size distribution curves of the BiVO_4_ sample synthesized using PAA.

The polyacrylic acid (PAA) was selected as an alternative polymer to replace PEI, in order to investigate whether the mesoporous single-crystalline BiVO_4_ could still be synthesized and to further elucidate the specific role of polymer functionalities in this assembly strategy. As shown in Figure S12, the BiVO_4_ sample synthesized using PAA also exhibits a distinct hysteresis loop characteristic of mesoporous structure, with a specific surface area of 20 m^2^/g, a pore size of 26 nm, and a pore volume of 0.098 cm^3^/g. This indicates that PAA can fulfill the role similar to that of PEI within the present synthesis strategy. We attribute this primarily to the abundant carboxyl groups of PAA, which act as strong hydrogen-bond acceptors and exhibit considerable coordination capability. These functions resemble those of PEI in the polymer-intercalated modulation assembly strategy, where it interacts with acetate ions and metal precursors via hydrogen bonding and coordination. Consequently, the PAA, acetate ions and metal precursors can co-assemble into the inorganic-organic composite, ultimately leading to the formation of mesoporous single-crystalline BiVO_4_.


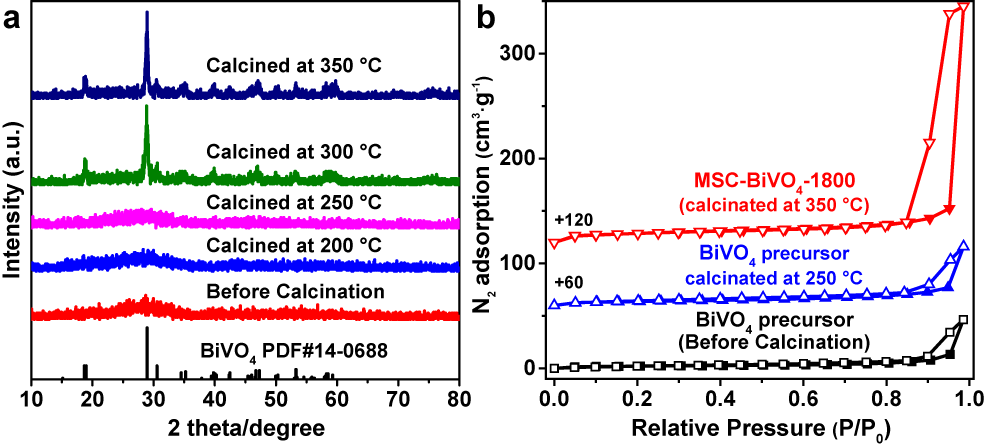


Figure S13. (a) XRD patterns and (b) N_2_ adsorption-desorption isotherms of the samples synthesized with different calcination temperatures.

In order to further elucidate the synthetic mechanism, XRD and N_2_ adsorption-desorption analyses were performed on the samples calcined at different temperatures. As shown in Figure S13a, negligible crystallization occurs in the BiVO_4_ precursor at calcination temperatures below 300 °C, with the samples remaining amorphous. When the temperature reaches 300 °C or above, the crystal structure essentially forms, corresponding to monoclinic BiVO_4_. This confirms that crystallization primarily initiates above 300 °C. Figure S13b indicates that the specific surface areas (8 and 13 m^2^/g, respectively) and pore volumes (0.049 and 0.062 cm^3^/g, respectively) of the BiVO_4_ precursor and its sample calcined at 250 °C are significantly lower than those of MSC-BiVO_4_-1800 (31 m^2^/g and 0.326 cm^3^/g). This suggests that washing and low-temperature calcination remove only trace of PEI, and create some pores. While most PEI remains within the pores, thereby supporting the mesoporous structure and preventing its collapse during the subsequent vigorous crystallization. Only when the calcination temperature is increased to 350 °C and crystallization is largely complete does the mesoporous structure finally form. The TG curve in Figure 3d also shows that significant weight loss occurs mainly after 300 °C, further validating the trends observed in the above N_2_ adsorption-desorption measurements. Based on the above, the sequence and mechanism of crystallization and pore formation during the calcination process were further elucidated, while also demonstrating the critical role of highly thermally stable PEI as a “porogenic agent” in the polymer-intercalated modulation assembly strategy.


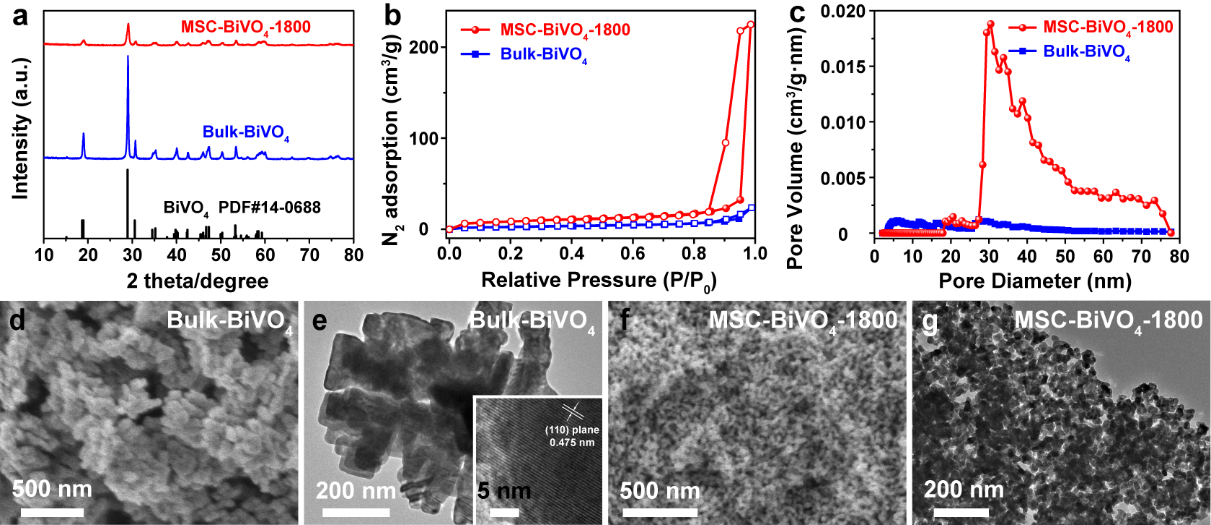


Figure S14. The crystal structure and pore structure characterizations of the photocatalysts (MSC-BiVO_4_-1800 and Bulk-BiVO_4_): (a) XRD patterns, (b) N_2_ adsorption-desorption isotherms and (c) corresponding pore size distribution curves, (d, e, inset of e) SEM, TEM and HRTEM images of Bulk-BiVO_4_, (f) SEM and (g) TEM images of MSC-BiVO_4_-1800.


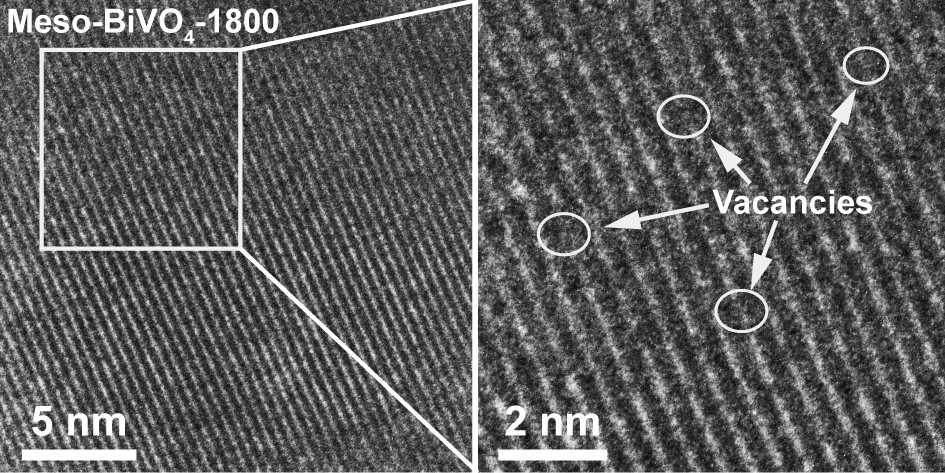


Figure S15. The different magnification HRTEM images of MSC-BiVO_4_-1800.





Figure S16. XPS V 2p spectra of MSC-BiVO_4_-1800 and Bulk-BiVO_4_.


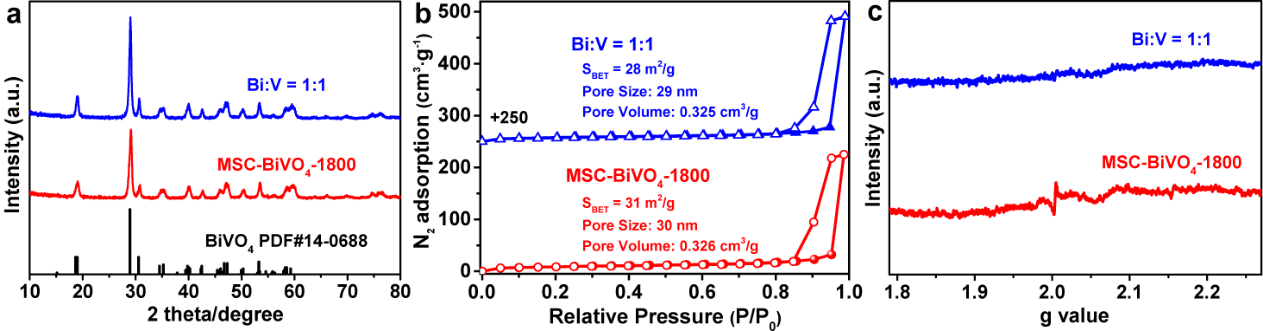


Figure S17. The characterizations and photocatalytic performance of MSC-BiVO_4_-1800 and the sample synthesized with 1:1 of Bi:V ratio: (a) XRD patterns, (b) N_2_ adsorption-desorption isotherms, (c) EPR spectra.

To investigate the effect of the non-stoichiometric precursor ratio (Bi:V = 1:0.8) on the structural properties and photocatalytic performance of MSC-BiVO_4_-1800, we synthesized a control sample with a stoichiometric Bi:V ratio of 1:1 and performed a comparative analysis of both samples (as shown in Figure S17). The XRD patterns (Figure S17a) confirm that both samples crystallize in the identical phase of monoclinic BiVO_4_, demonstrating that the slightly V‑deficient feeding ratio does not alter the fundamental crystal structure. N_2_ adsorption-desorption analysis (Figure S17b) further reveals that the sample synthesized with reduced V content possesses a slightly increased specific surface area and enlarged pore size, which is beneficial for reactant adsorption and mass transfer. Most notably, EPR spectra (Figure S17c) shows a characteristic signal for V vacancies in MSC-BiVO_4_-1800, which is absent in the sample synthesized with stoichiometric Bi:V ratio of 1:1. This provides direct evidence that the reduced V precursor content effectively promotes the formation of V vacancies.

Collectively, these results resolve the apparent discrepancy: the non‑stoichiometric feeding does not produce an off‑stoichiometric sample, but rather introduces V vacancies within the well‑formed BiVO_4_ framework. The above various characterization results confirm that the deviation from the nominal 1:1 ratio is a deliberate and effective synthetic lever for creating the active V vacancy microenvironment, which is vital for enhancing the photocatalytic performance.


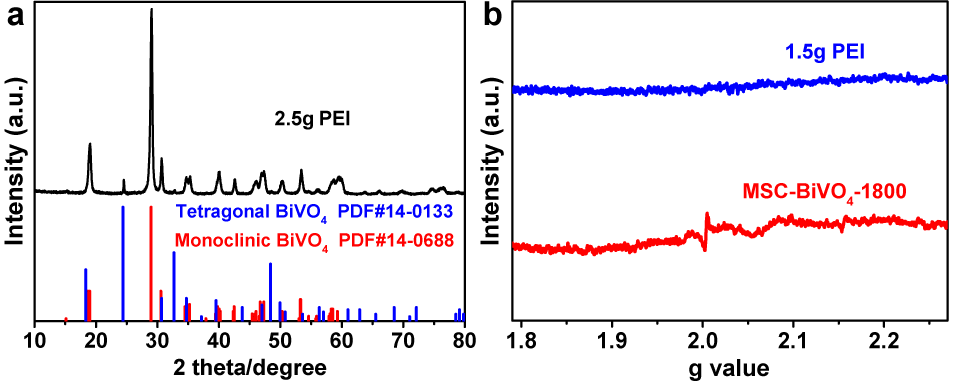


Figure S18. (a) XRD pattern of the MSC-BiVO_4_ sample synthesized with increased PEI amount of 2.5g, (b) EPR spectra of MSC-BiVO_4_-1800 and the sample synthesized with decreased PEI amount of 1.5g.

The equilibrium relationship between V vacancy concentration and mesopore proportion was investigated by synthesizing BiVO_4_ samples with varying amounts of PEI. As shown in Figure S18a, when the amount of PEI was increased to 2.5g, the excessive introduction of PEI altered the microscopic interactions and co-assembly process among the metal precursors, acetate ions, and PEI, leading to a change in the crystal structure. Consequently, a homojunction BiVO_4_ containing both monoclinic and tetragonal phases was synthesized, rather than a pure monoclinic BiVO_4_. Therefore, it is difficult to further investigate the effect of the increase in the proportion of mesopores on the concentration of V vacancies by increasing the amount of PEI. When the PEI amount was reduced to 1.5g, the mesopore proportion decreased, accompanied by a reduction in specific surface area and pore volume to 21 m^2^/g and 0.277 cm^3^/g, respectively (Table S2). However, its EPR spectra (Figure S18b) showed no significant signal, indicating an extremely low V vacancy concentration. In summary, we conclude that MSC-BiVO_4_-1800 likely possesses an appropriate concentration of V vacancies. Moreover, for this synthesis system, precise regulation of V vacancy concentration by modulating the mesopore proportion remains challenging, which may be an important direction for our future research.

Based on the above findings and analysis, we speculate that the V vacancy microenvironment constructed in this work exhibits a relatively low vacancy concentration, which may not introduce a significant number of defect sites capable of promoting charge carrier recombination. This inference is supported by Figures 6a-c and S23, which demonstrate that MSC-BiVO_4_-1800 significantly enhances the separation of photogenerated carriers.


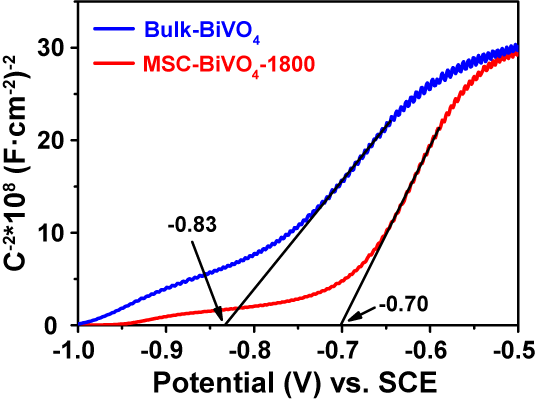


Figure S19. The Mott-Schottky plots of the photocatalysts.

The conduction band (CB) and valence band (VB) potentials were measured and analyzed to investigate both photocatalysts from a thermodynamic perspective. As shown in Figure S19, the flat band potentials of MSC-BiVO_4_-1800 and Bulk‑BiVO_4_ *vs.* saturated calomel electrode (SCE) are -0.70 V and -0.83 V, respectively. From these values, the corresponding CB potentials *vs.* normal hydrogen electrode (NHE) are calculated to be -0.46  V and -0.59  V. Both CB potentials are significantly more negative than the redox potential of E(O_2_/•O_2_^-^) = -0.33 V vs. NHE, indicating that, thermodynamically, both photocatalysts are capable for reducing O_2_ to generate •O_2_^-^. Furthermore, based on the bandgaps derived from Figure 4d (2.46 eV for MSC-BiVO_4_-1800 and 2.55 eV for Bulk‑BiVO_4_) and the obtained CB potentials, the VB potentials of both photocatalysts are calculated to be 2.00 V and 1.96 V vs. NHE, respectively. The VB potential of MSC-BiVO_4_-1800 (2.00 V) is higher than the redox potential E(BA/BAD) = 1.98 V vs. NHE, whereas that of Bulk‑BiVO_4_ (1.96 V) is slightly lower. This implies that, in principle and from a thermodynamic perspective, the photogenerated h^+^ in MSC-BiVO_4_-1800 can drive the selective oxidation of BA, while those in Bulk‑BiVO_4_ likely cannot. Consequently, the bandgap structure analysis suggests that MSC-BiVO_4_-1800 is thermodynamically more favorable for promoting the photocatalytic selective oxidation of BA.


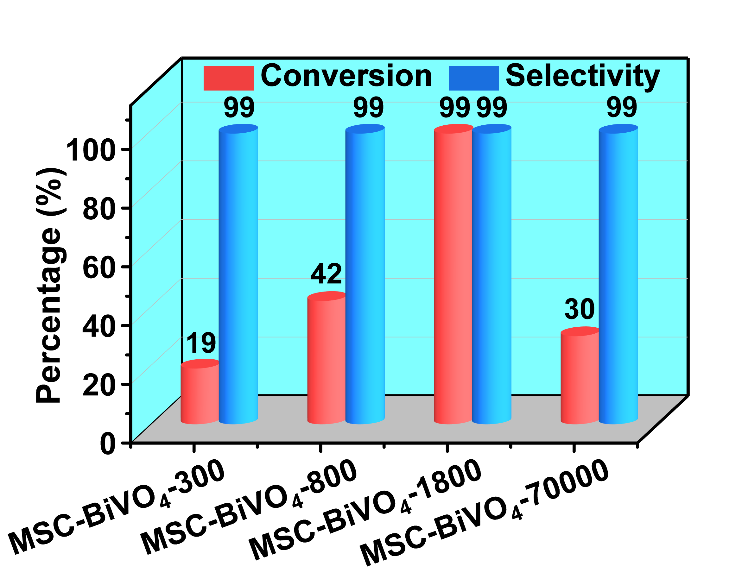


Figure S20. Photocatalytic performance (conversion and selectivity) of MSC-BiVO_4_-300, MSC-BiVO_4_-800, MSC-BiVO_4_-1800 and MSC-BiVO_4_-70000 after 5 h irradiation.


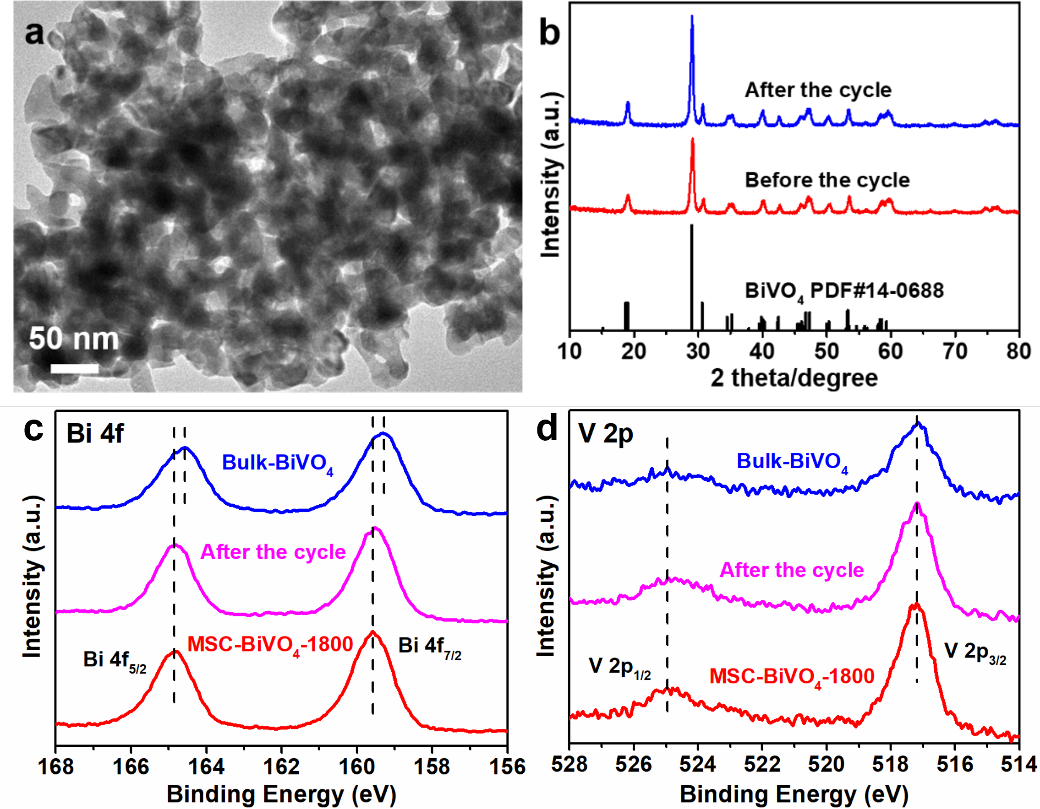


Figure S21. (a) TEM image of MSC-BiVO_4_-1800 after the cycle; (b) XRD patterns of MSC-BiVO_4_-1800 before and after the cycle; (c) XPS Bi 4f spectra and (d) XPS V2p spectra of MSC-BiVO_4_-1800 before and after the cycle, and Bulk- BiVO_4_.


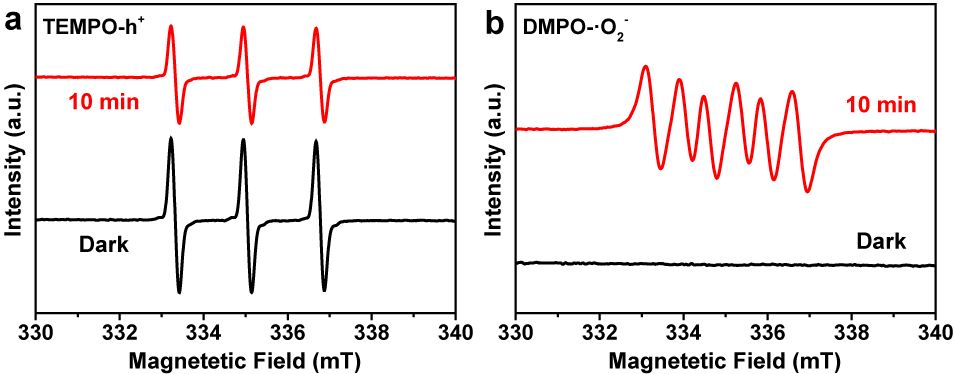


Figure S22. The *quasi in-situ* ESR spectra of (a) TEMPO-h^+^ and (b) DMPO-•O_2_^-^ generated by MSC-BiVO_4_-1800 under dark and simulated sunlight irradiation conditions.


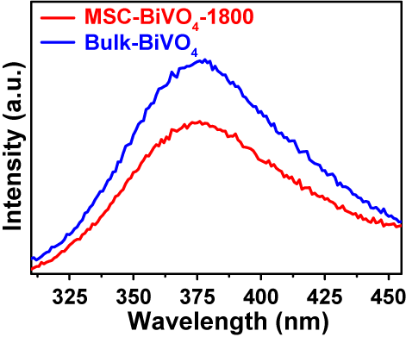


Figure S23. The PL spectra of the photocatalysts.

**Section 3. Supplementary Tables**

Table S1. Specific surface area and pore structure properties of samples synthesized with different amount of HOAc.

| Sample | S_BET_ (m^2^/g) | Pore size (nm) | Total pore volume (cm^3^/g) |
| --- | --- | --- | --- |
| 0 mmol HOAc | 38 | 26 | 0.179 |
| 35 mmol HOAc | 26 | 27 | 0.119 |
| 70 mmol HOAc | 31 | 29 | 0.326 |

Table S2. The specific surface area and pore structure properties of mesoporous BiVO_4_ synthesized with different amount of PEI.

| Sample | S_BET_ (m^2^/g) | Pore size (nm) | Total pore volume (cm^3^/g) |
| --- | --- | --- | --- |
| BiVO_4_-0g PEI | 3 | 4 | 0.007 |
| BiVO_4_-0.25g PEI | 8 | 4 | 0.027 |
| BiVO_4_-0.5g PEI | 3 | 13 | 0.010 |
| BiVO_4_-1g PEI | 29 | 27 | 0.167 |
| BiVO_4_-1.5g PEI | 21 | 31 | 0.277 |
| BiVO_4_-2.1g PEI | 31 | 29 | 0.326 |

Table S3. Structural properties of MSC-BiVO_4_ samples synthesized with different PEI molecular weights (Mw. 300-70000).

| Sample | S_BET_ (m^2^/g) | Pore size (nm) | V_T_ (cm^3^/g) |
| --- | --- | --- | --- |
| MSC-BiVO_4_-300 | 22 | 26 | 0.078 |
| MSC-BiVO_4_-800 | 18 | 29 | 0.100 |
| MSC-BiVO_4_-1800 | 31 | 30 | 0.326 |
| MSC-BiVO_4_-70000 | 19 | 29 | 0.154 |

Table S4. The carbon content of the samples measured by CHN analysis.

| Sample | Carbon content |
| --- | --- |
| MSC-BiVO_4_-300 | 0.29% |
| MSC-BiVO_4_-800 | 0.27% |
| MSC-BiVO_4_-1800 | 0.24% |
| MSC-BiVO_4_-70000 | 0.35% |
| Bulk-BiVO_4_ | 0.05% |
| MSC-BiVO_4_-0g PEI | 0.12% |
| MSC-BiVO_4_-0.5g PEI | 0.24% |
| MSC-BiVO_4_-1g PEI | 0.33% |
| MSC-BiVO_4_-1.5g PEI | 0.20% |

Table S5. The detailed data of the MSC-BiVO_4_-1800 and Bulk-BiVO_4_ photocatalysts.

|  | | MSC-BiVO_4_-1800 | Bulk-BiVO_4_ |
| --- | --- | --- | --- |
| Specific surface area (m^2^/g) | | 31 | 9 |
| Pore size (nm) | | 30 | 27 |
| Pore volume (cm^3^/g) | | 0.326 | 0.034 |
| Particle size (nm) | | 16.2 | 30.2 |
| n_V_:n_Bi_ | ICP | 0.84 | 0.95 |
|  | XRF | 0.76 | 0.81 |

Table S6. The photocatalytic selective oxidation performance of BA for the BiVO_4_ samples synthesized with different methods.

| Photocatalyst | Conversion | Selectivity |
| --- | --- | --- |
| MSC-BiVO_4_-1800 | 99% | 99% |
| MSC-BiVO_4_-1800-Bi:V=1:1 | 70% | 99% |
| MSC-BiVO_4_-300 | 19% | 99% |
| MSC-BiVO_4_-800 | 42% | 99% |
| MSC-BiVO_4_-70000 | 30% | 99% |
| MSC-BiVO_4_-0g PEI | 25% | 99% |
| Bulk-BiVO_4_ | 19% | 99% |

Reaction condition: catalyst (30 mg), substrate (16.7 mM), acetonitrile (3 mL), 1 atm O_2_, Xe lamp (350-780 nm) irradiation for 5 h.

To provide direct performance comparisons and highlight the advantages of prepared MSC-BiVO_4_, the photocatalytic performance of different control samples is summarized in the Table S6. While all samples maintained high selectivity (99%), their conversion rates varied significantly and were all substantially lower than that of MSC-BiVO_4_-1800 (99%):

Mesoporous single-crystalline BiVO_4_ without V vacancies: 70% conversion (Table S6). Mesoporous single-crystalline BiVO_4_ with different pore structures (synthesized with different molecular weights of PEI): 19-42% conversion (Figure S20 and Table S6). Non-porous single-crystalline BiVO_4_ (synthesized without PEI addition): 25% conversion (Table S6). Non-porous polycrystalline commercial Bulk-BiVO_4_: 19% conversion (Figure 5b and Table S6).

This systematic comparison clearly validates the critical, synergistic role of the V vacancy microenvironment, mesoporous structure, and single-crystalline nature in achieving superior photocatalytic performance for MSC-BiVO_4_-1800. The lower conversion of each control sample emphasizes that the superior photocatalytic performance of MSC-BiVO_4_-1800 arises from the unique integration of above three structural features enabled by the polymer-intercalated modulation assembly strategy.

Table S7. The photocatalytic selective oxidation performance of different aromatic alcohols by MSC-BiVO_4_-1800 photocatalyst.

| Entry | Substrate | Reaction time | Product | Conv. | Sel. |
| --- | --- | --- | --- | --- | --- |
| 1 |  | 3 h |  | 99% | 99% |
| 2 |  | 3 h |  | 99% | 99% |
| 3 |  | 3 h |  | 99% | 99% |
| 4 |  | 8 h |  | 99% | 99% |
| 5 |  | 5 h |  | 99% | 99% |
| 6 |  | 4 h |  | 99% | 99% |
| 7 |  | 3 h |  | 99% | 99% |
| 8 |  | 3 h |  | 99% | 99% |

Reaction condition: catalyst (30 mg), substrate (16.7 mM), acetonitrile (3 mL), 1 atm O_2_, Xe lamp (350-780 nm) irradiation.

Table S8. The detailed data of TRPL spectra for MSC-BiVO_4_-1800 and Bulk-BiVO_4_.

| Parameter | MSC-BiVO_4_-1800 | Bulk-BiVO_4_ |
| --- | --- | --- |
| τ_1_ | 2.411 ns | 1.445 ns |
| τ_2_ | 11.23 ns | 7.79 ns |
| B_1_ | 2221.308 | 2513.201 |
| B_2_ | 427.927 | 566.98 |
| τ | 5.311 ns | 4.275 ns |
